# Interindustry linkages of prices—Analysis of Japan’s deflation

**DOI:** 10.1371/journal.pone.0228026

**Published:** 2020-02-13

**Authors:** Yuichi Kichikawa, Hiroshi Iyetomi, Hideaki Aoyama, Yoshi Fujiwara, Hiroshi Yoshikawa

**Affiliations:** 1 Faculty of Science, Niigata University, Niigata, Japan; 2 Department of Mathematics, Niigata University, Niigata, Japan; 3 GSAIS, Kyoto University, Kyoto, Japan; 4 RIETI, Tokyo, Japan; 5 Graduate School of Simulations, University of Hyogo, Kobe, Japan; 6 Faculty of Economics, Rissho University, Tokyo, Japan; RIKEN, JAPAN

## Abstract

The interactions among macroprices with leads and lags play a significant role in explaining the behavior of an aggregate price index. Thus, to understand inflation and deflation, it is essential to explore the mechanism according to which these macroprices interact with each other. On the basis of a new method, we show that, irrespective of the sources of shocks, a robust flow of changes occurs in domestic prices from upstream to downstream. Moreover, we demonstrate that macroprices change in clusters, and we identify these clusters. Firms are not symmetric. Overall, our analysis suggests that the inertia arising from input/output linkages in production explains the behavior of aggregate prices.

## Introduction

Price behavior is of primary importance in economics. In macroeconomics, inflation, together with unemployment, is one of the most important issues driving policy decisions. More recently, however, deflation has started to be regarded as a threat to the macroeconomy. In the late 1990’s, Japan was the first of the advanced countries to become trapped in deflation, amidst which the country faced the zero interest bound. Krugman [1], instead of the traditional interest policy, advanced the use of quantitative easing (QE) coupled with a firmly committed inflation target as a possible remedy. Many others have elaborated on the idea [2–5]. Today, most central banks are indeed committed to an explicit inflation target such as an annual two-percent increase in the consumer price index (CPI). Facing the zero interest bound, they struggle against deflation by resorting to unconventional monetary policies such as quantitative easing, forward guidance, and negative interest rates. The efficacy of these policies with respect to price control depends, of course, on how prices are determined.

In macroeconomic theory, prices are said to be “sticky.” In fact, in modern dynamic stochastic general equilibrium (DSGE) models, monetary policy is effective to the extent that prices are sticky. A number of theories attempting to explain sticky prices have been proposed: the Taylor–Calvo model of desynchronized staggered wage/price changes [6] and menu cost models [7], to name a few. Based on these micro-foundations, the standard framework for understanding the role of monetary policy is the New Keynesian Phillips curve (NKPC).

The key property of the NKPC is that inflation is primarily a forward-looking process. That is, expectations on future inflation largely determine current inflation. This justifies recent emphasis on expectations management and communications as tools of monetary policy. A large amount of literature exists on the NKPC. However, after a lengthy survey of the literature, Mavroeidis, Plagborg-Møller, and Stock [8] reached quite a disappointing conclusion. Namely, their major finding was that estimation of the NKPC using macroeconomic data are subject to a severely weak instruments problem. Indeed, they found that “the evidence is consistent both with the view that expectations matter a lot, as well as with the opposite view that they matter very little”. They thus concluded that identification of the NKPC is too weak to warrant research on conceptually minor extensions. The traditional analysis based on macroeconomic data has its clear limitations.

Meanwhile, recent empirical studies on microprice-setting as surveyed by Klenow and Malin [9] have uncovered the hitherto little known dynamics of microprices (Ref. [10], for Japan). Bils and Klenow [11], for example, by examining the frequency of price changes for 350 categories of goods and services, demonstrated that half of the prices remain valid for 5.5 months or less. Their findings seemed to suggest that individual prices are actually not rigid. There are substantial differences across goods, however; prices of raw materials and foodstuff are flexible whereas those of services are less flexible. The fact is well known. A long time ago, Kalecki [12] proposed a two-sector model of prices: The prices of raw materials and foodstuff are determined flexibly by market forces of supply and demand whereas prices of most manufactured products and services are determined by suppliers based on their production costs [13]. Today, central banks are committed to inflation targeting with respect to the “core” CPI which excludes the prices of foodstuff and energy.

Studies of microprices provide useful information. However, deflation and inflation are nothing but changes in the aggregate price *over time* whereas the existing theoretical literature on microprices focuses mostly on *cross-sectional* differences among microprices. The work of Berger and Vavra [14] fills this void. They demonstrate that the U.S. price distribution is not constant but varies with time. Specifically, they show that the frequency and variance of price changes are counter-cyclical.

Following their lead, we explore the dynamics of prices. In particular, we focus on interactions of prices with leads and lags. They play a significant role in explaining the behavior of the aggregate price index. Thus, to understand inflation and deflation, it is essential to explore the mechanism of interactions among prices. Resorting to a new method, we show that irrespective of the sources of shocks, there exists a robust flow of changes in domestic prices from upstream to downstream. Moreover, we demonstrate that prices change in clusters, and identify these clusters. Overall, our analysis suggests that the inertia arising from input/output linkages in production explains the behavior of aggregate prices.

We first briefly summarize Japan’s inflation and deflation for the last thirty years in the next section. In the third section, we study the prices of 80 goods and services and 7 macroeconomic variables for Japan. To uncover the dynamics of prices with leads and lags, we resort to a new method named Complex Hilbert Principal Component Analysis (CHPCA). Our analysis identifies two driving forces, one external and the other domestic. However, irrespective of the nature of a shock, a robust propagation mechanism exists for domestic prices. Then, in the following section, we demonstrate that domestic prices change in clusters, using a new method named the Hodge decomposition and percolation theory. The last section offers our concluding remarks.

## The behavior of prices

Beginning in the late 1990’s, Japan experienced more than a decade of notorious deflation. Amid the deflation, the Bank of Japan (BoJ) faced the zero interest bound. The U.S. Federal Reserve and the European Central Bank eventually followed suit. The world paid considerable attention to this phenomenon; even the word “Japanization” was coined.

In the early stage, most macroeconomists believed that sizable quantitative easing would turn deflation into mild deflation [1]. However, solving the problem was discovered to be more difficult than that. For example, the BoJ has pursued unprecedentedly sizable QE by increasing the monetary base from 40 trillion yen as of December, 2012 to 400 trillion yen by the end of 2017. Despite their efforts, the rate of change of the consumer price remains only 0.4 percent, falling short of the target rate of 2%. In the U.S., the chair of the Federal Reserve, Janet Yellen, regarded “low price” as a kind of puzzle. Clearly, further analysis of prices is necessary.

**Fig 1:**
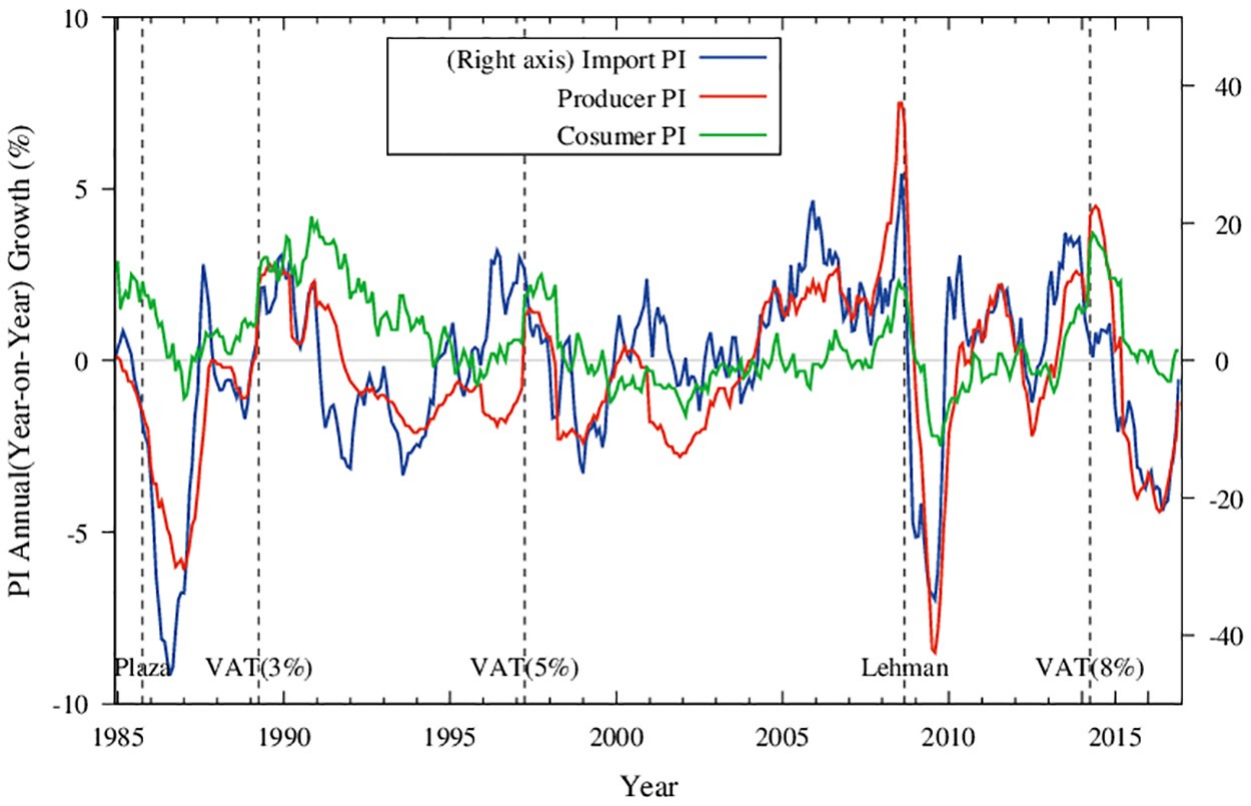
Overview of the aggregate prices. Time-series of monthly price indices (PI) for Import PI (blue and right-axis), Producer PI (red), and Consumer PI (green) from 1980 up to present. Dashed vertical lines correspond to the three months in which VAT was raised, namely, April of 1989, 1997 and 2014 (VAT 3%, 5%, 8% respectively), and September 2008 in which the Lehman Brothers went into bankruptcy.

Following [14], we studied three kinds of prices. Fig 1 shows the aggregates of these prices, namely, the import price, producer price, and consumer price of Japan for three decades. Plainly, the import price is much more volatile than both the producer and consumer prices (note that a different scale is used to indicate changes in the import price on the axis on the right). In fact, import prices often declined substantially. For example, in the fall of 1985, the Plaza Agreement was reached, and the yen started appreciating from 240 yen per dollar to 120 yen per dollar. As a consequence, the aggregate import price in terms of yen was reduced to half of its former value. In parallel, the producer price also declined by 7%. The decline in the price of crude oil also affected Japan’s import price substantially as observed for the years 2009 and 2016. However, we are primarily interested in the consumer price simply because it is the target for the central bank’s monetary policy.

## Dynamics of prices and macroeconomic variables

Inflation and deflation reflect changes in the aggregate price index, and this index comprises microprices. Obviously, understanding the behavior of the aggregate price means it is essential to understand the nature of (1) interactions among individual prices and (2) their relationships with changes in the macroeconomic variables. In this section, we present our new analytical method named Complex Hilbert Principal Component Analysis.

In what follows, we use data of individual prices specific to industrial sectors. An individual price is actually defined by a weighted average of each of the goods and services in the sector. In this sense, an individual price in an industrial sector can be regarded as a *mesoscopic* variable, the interaction and change of which is of our interest here. Therefore, we refer to such mesoscopic variables as “meso” prices.

### Data

We use the Japanese monthly data of the following 80 mesoprices for the period January 1985 through December 2016:

- Import Price Index (IPI), compiled by the Bank of Japan (BoJ) consists of “prices of ⋯ imports at the stage of entry into Japan” [15]. 10 prices, ID = 1–10 in Table 1.
- Producer Price Index (PPI) compiled by the BoJ, “surveys the prices of goods traded among companies, specifically domestically produced goods for domestic markets, mainly at the stage of shipment from producers and partly from wholesalers” [15]. – 2015 base Intermediate classification, excluding consumption tax, 23 prices, ID = 11–33 in Table 1.
- Consumer Price Index (CPI) compiled by the Statistics Bureau of the Ministry of Internal Affairs and Communications [16]. – 2015 base Intermediate classification, Statistics Bureau of Japan, 47 prices, ID = 34–80 in Table 2.

**Table 1:** List of prices in the PPI and IPI categories.

ID	IPI
1	Foodstuffs & feedstuffs
2	Textiles
3	Metals & related products
4	Wood, lumber & related products
5	Petroleum, coal & natural gas
6	Chemicals & related products
7	General purpose, production & business oriented machinery
8	Electric & electronic products
9	Transportation equipment
10	Other primary products & manufactured goods
ID	PPI
11	Food, beverages, tobacco & feedstuffs
12	Textile products
13	Lumber & wood products
14	Pulp, paper & related products
15	Chemicals & related products
16	Petroleum & coal products
17	Plastic products
18	Ceramic, stone & clay products
19	Iron & steel
20	Nonferrous metals
21	Metal products
22	General purpose machinery
23	Production machinery
24	Business oriented machinery
25	Electronic components & devices
26	Electrical machinery & equipment
27	Information & communications equipment
28	Transportation equipment
29	Other manufacturing industry products
30	Agriculture, forestry & fishery products
31	Minerals
32	Electric power, gas & water
33	Scrap & waste

**Table 2:** List of prices in the CPI category.

ID	CPI
34	Cereals
35	Fish & seafood
36	Meats
37	Dairy products & eggs
38	Vegetables & seaweeds
39	Fruits
40	Oils, fats & seasonings
41	Cakes & candies
42	Cooked food
43	Beverages
44	Alcoholic beverages
45	Meals outside the home
46	Rent
47	Repairs & maintenance
48	Electricity
49	Gas
50	Other fuel & light
51	Water & sewerage charges
52	Household durable goods
53	Interior furnishings
54	Bedding
55	Domestic utensils
56	Domestic non-durable goods
57	Domestic services
58	Clothes
59	Shirts, sweaters & underwear
60	Footwear
61	Other clothing
62	Services related to clothing
63	Medicines & health fortification
64	Medical supplies & appliances
65	Medical services
66	Public transportation
67	Private transportation
68	Communication
69	School fees
70	School textbooks & reference books for study
71	Tutorial fees
72	Recreational durable goods
73	Recreational goods
74	Books & other reading materials
75	Recreational services
76	Personal care services
77	Toilet articles
78	Personal effects
79	Tobacco
80	Other miscellaneous

We also used the following seven monthly macroeconomic variables:

81 Japanese Yen to US Dollar Exchange Rate (JPY/USD)–Tokyo market, monthly average, Bank of Japan.
82–84 Index of Business Condition (Leading, Coincident, Lagging)–Composite Index 2015 base, outlier processed, Cabinet Office, Government of Japan.
85 Money Stock (M2)—Bank of Japan.
86 Monetary Base.—Bank of Japan
87 Nominal wage (Contractual cash earnings (Manufacturing)),–Health, Labour and Welfare Ministry, Japan.

All together, we used time series of 87 variables consisting of 80 mesoprices and 7 macroeconomic variables for a period of 384 months.

The heterogeneity of mesoprices found in the existing literature can be easily confirmed for the Japanese data we analyzed. Table 3 lists the mean duration *d* (in months) of the period during which individual prices remained unchanged for 39 groups of goods and services. The table also provides the values of λ, the monthly frequency, or the probability that the price would change within a month (not directly observed). Assuming that the prices could change at any instance in time with a constant probability, a simple Poisson process leads to the result that *d* is equal to −1/ln(1 − λ). Given the value of *d*, the values for λ in the table are estimated by this formula.

**Table 3:** Properties of meso prices.

ID	Classification of sector	#Goods	Months	Freq
IPI—Import PI				
01	Foodstuffs & feedstuffs	20	1.02	62.34
02	Textiles	7	1.17	57.56
03	Metals & related products	20	1.06	61.08
04	Wood, lumber & related products	3	1.01	62.73
05	Petroleum, coal & natural gas	8	1.03	62.16
06	Chemicals & related products	10	1.41	50.69
07	General purpose, production & business oriented machinery	2	1.11	59.50
08	Electric & electronic products	3	1.09	59.90
09	Other primary products & manufactured goods	11	1.11	59.32
—	All	84	1.11	59.48
PPI—Producer PI				
01	Food, beverages, tobacco & feedstuffs	83	2.64	31.56
02	Textile products	20	7.79	12.05
03	Lumber & wood products	11	3.37	25.67
04	Pulp, paper & related products	25	3.13	27.34
05	Chemicals & related products	64	5.82	15.78
06	Petroleum & coal products	11	1.92	40.56
07	Plastic products	14	3.61	24.19
08	Ceramic, stone & clay products	30	4.21	21.15
09	Iron & steel	27	4.17	21.33
10	Nonferrous metals	21	1.47	49.38
11	Metal products	27	4.07	21.78
12	General purpose machinery	21	4.92	18.38
13	Production machinery	21	3.16	27.12
14	Business oriented machinery	14	6.21	14.88
15	Electronic components & devices	8	2.30	35.31
16	Electrical machinery & equipment	25	3.37	25.68
17	Information & communications equipment	3	2.56	32.29
18	Transportation equipment	11	9.29	10.21
19	Other manufacturing industry products	25	7.27	12.85
20	Agriculture, forestry & fishery products	18	4.76	18.97
21	Minerals	3	9.96	9.56
22	Electric power, gas & water	3	8.93	10.60
23	Scrap & waste	5	1.11	59.51
—	All	490	4.31	20.70
CPI—Consumer PI				
01	Food	151	1.33	52.98
02	Housing	13	1.72	44.18
03	Fuel, Light & Water Charges	6	2.29	35.40
04	Furniture & Household Utensils	33	1.21	56.19
05	Clothes & Footwear	58	1.58	46.94
06	Medical Care	14	2.48	33.15
07	Transportation & Communication	23	18.74	5.20
08	Education	11	9.96	9.55
09	Culture & Recreation	33	6.22	14.86
10	Miscellaneous	24	3.84	22.94
—	All	366	3.39	25.55

List of IDs, classification of sectors, number of goods, and durations and frequencies of price changes for the commodities of the IPI, PPI, and CPI. The sectors for the IPI and PPI correspond to the major groups based on the BOJ datasets. Those for the CPI are classified by the authors and are partially based on the original classification and identities. Months is the mean duration between price changes, denoted by *d*. Freq is the constant monthly frequency of price changes or probability (in percent) of the price changing in a month, λ, estimated from *d* based on a simple assumption of the Poisson process, i.e., by *d* = −1/ln(1 − λ).

The mean duration varies from 10 months for business machinery and transportation equipment to one month for food, clothing, and most imported goods and materials. Chemicals in the PPI and services in the CPI have intermediary mean durations of 6 months. On the whole, the prices of imported goods and materials are highly flexible. They are broadly consistent with previous results on prices of other countries [9].

### Method—Complex Hilbert Principal Component Analysis

The interactions and co-movements among individual prices and macroeconomic variables can be studied by examining their correlations. One may consider ordinary principal component analysis (PCA) or factor analysis, both of which are widely used in economics as well as in other disciplines, appropriate to uncover the “hidden” factors responsible for generating co-movements of multi-variables. This would, however, present a serious problem because there exist significant *leads and lags* among the variables we analyze. In the data considered in our study, it is most natural to expect that some prices are affected by changes in some other prices, with significant lags. Therefore, we would need to extend PCA to enable it to accommodate time lead/lag. If there were only two time series, we could use cross-spectral analysis to identify the lead-lag relationship between them. In reality, however, we have more than two time series and aim to examine their collective motions, in which case the conventional method is not valid.

Motivated to address these shortcomings, we decided to use Complex Hilbert Principal Component Analysis (CHPCA), which solves the problem in a unified manner. CHPCA allows us to conduct one calculation for the entire set to extract significant co-movements with leads and lags which often span the entire set. This method has been successfully applied to various subjects ranging from meteorology/climatology to signal processing to finance and economics ([17–21]). However, the method remains relatively unknown in economics. In the following, we briefly explain the CHPCA method, which consists of the following steps.

1. We consider the logarithmic change in the mesoprice *P*_*α*_(*t*) at time *t*, i.e.,
  $$\begin{array}{c} x_{\alpha }\left(t\right)=\log\frac{P_{\alpha }\left(t\right)}{P_{\alpha }\left(t-1\right)}. \end{array}$$ (1)
  We then standardize *x*_*α*_(*t*) by subtracting the sample mean and normalize by using the standard deviation of the sample. The time series thus obtained is denoted by ${\tilde{x}}_{\alpha }\left(t\right)$.
2. We complexify each time series, ${\tilde{x}}_{\alpha }\left(t\right)$. For this purpose, we decompose it into its Fourier components, and then replace sin(*ωt*) by *ie*^−*iωt*^ and cos(*ωt*) by *e*^−*iωt*^ in each component. Note that by this operation the original time series remains as the real part of the complexified time series. The resulting complex components rotate in a clock-wise direction on its complex plane. Let us denote the resulting complexified time series as ${\tilde{w}}_{\alpha }\left(t\right)$.
3. Next, we calculate the complex correlation coefficient
  $$\begin{array}{c} {\tilde{C}}_{\alpha \beta }:={\left\langle {\tilde{w}}_{\alpha }{\tilde{w}}_{\beta }^{*}\right\rangle }_{t}, \end{array}$$ (2)
  where 〈 ⋅ 〉_*t*_ denotes the average over time. The symbol * represents the complex conjugate. This complex correlation coefficient ${\tilde{C}}_{\alpha \beta }$ provides (1) the strength of the correlation between time-series *α* and *β* by its absolute value, and (2) the time delay between them by its phase. We then obtain the eigenvalues λ^(*n*)^ and the eigenvectors ***V***^(*n*)^ for $\tilde{C}=\left\{{\tilde{C}}_{\alpha \beta }\right\}$:
  $$\begin{array}{c} \tilde{C}\;V^{\left(n\right)}=\lambda^{\left(n\right)}V^{\left(n\right)}. \end{array}$$ (3)
  We note that ***V***^(*n*)†^***V***^(*m*)^ = *δ*_*nm*_ (where the symbol † represents the Hermitian conjugate, namely, the operation of transpose and the complex conjugate), and $\sum_{n=1}^{N}\lambda^{\left(n\right)}=N$ hold where *N* is the number of time series.
4. We identify significant eigenmodes that represent statistically significant co-movements (signals) by carrying out the significance test by simulation using Rotational Random Shuffling (RRS), which is a well-established significance test ([21, 22]). The difficulty of requiring the significance test for eigenvectors even arises for ordinary principal component (factor) analysis.

In this test, the null is eigenvalues that are obtained for randomized data. Thus, in this simulation, correlations between time series are destroyed by rotating each time series (with its head and the end joined) randomly and independently, after which the eigenvalues are calculated. This exercise is repeated a number of times to obtain the distribution of eigenvalues. Any eigenvalue above the distribution corresponding to the RRS is significant.

In the following subsection, we present the results we obtained for data comprising 80 prices and 7 macroeconomic variables. We demonstrate the effectiveness of the CHPCA in extracting the collective behavior of prices using synthesized data in Appendix A in S1 File.

### Results—Significance test of principal components

First, we compute the eigenvalues of the complex correlation matrix $\tilde{C}$ constructed from the price data, and then carry out a significance test for the principal components based on the RRS as null model.

**Fig 2:**
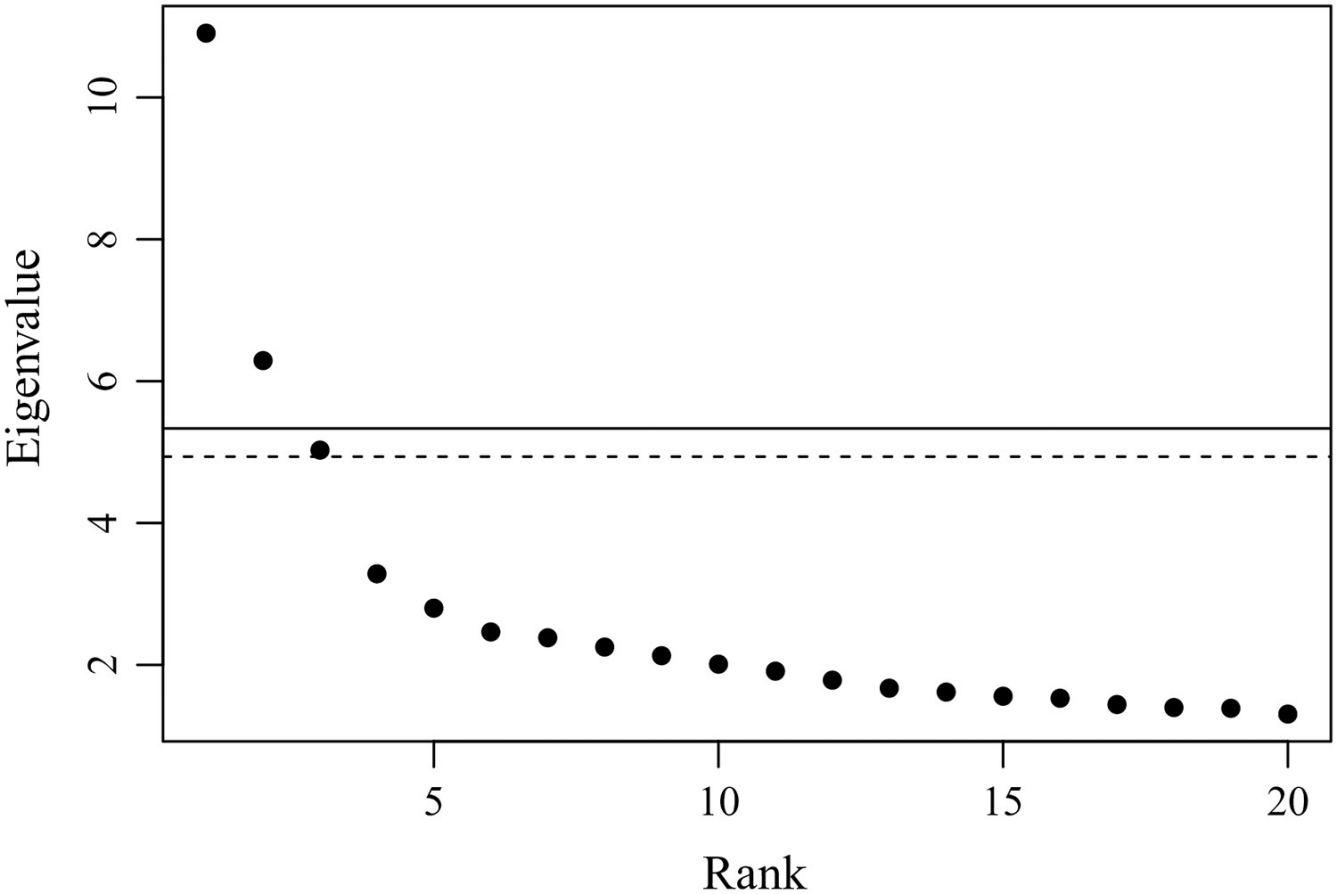
Eigenvalues obtained by the CHPCA with the RRS criterion. The points are the actual CHPCA eigenvalues. The solid horizontal line shows the upper limit of the largest eigenvalue predicted by the RRS simulation with 1000 samples at 3*σ* confidence level while the dashed line shows its average value; the largest and second largest eigenvalues are statistically meaningful.

Fig 2 compares the actual eigenvalues of the CHPCA with the results of the RRS simulation with 1,000 samples. Here we take the upper limit of the largest eigenvalue predicted by the RRS at the 3*σ* confidence level as a criterion for the significance test. In Fig 2, we observe that the two largest eigenvalues are significant. The eigenvectors associated with those eigenvalues are hence regarded as statistically significant correlations among individual prices.

In what follows, we focus on the first and second eigenmodes. We note that the basic properties of the eigenvectors do not depend on whether the seasonal adjustment is applied to the time-series data.

### Interpretation of the first and second eigenmodes

**Fig 3:**
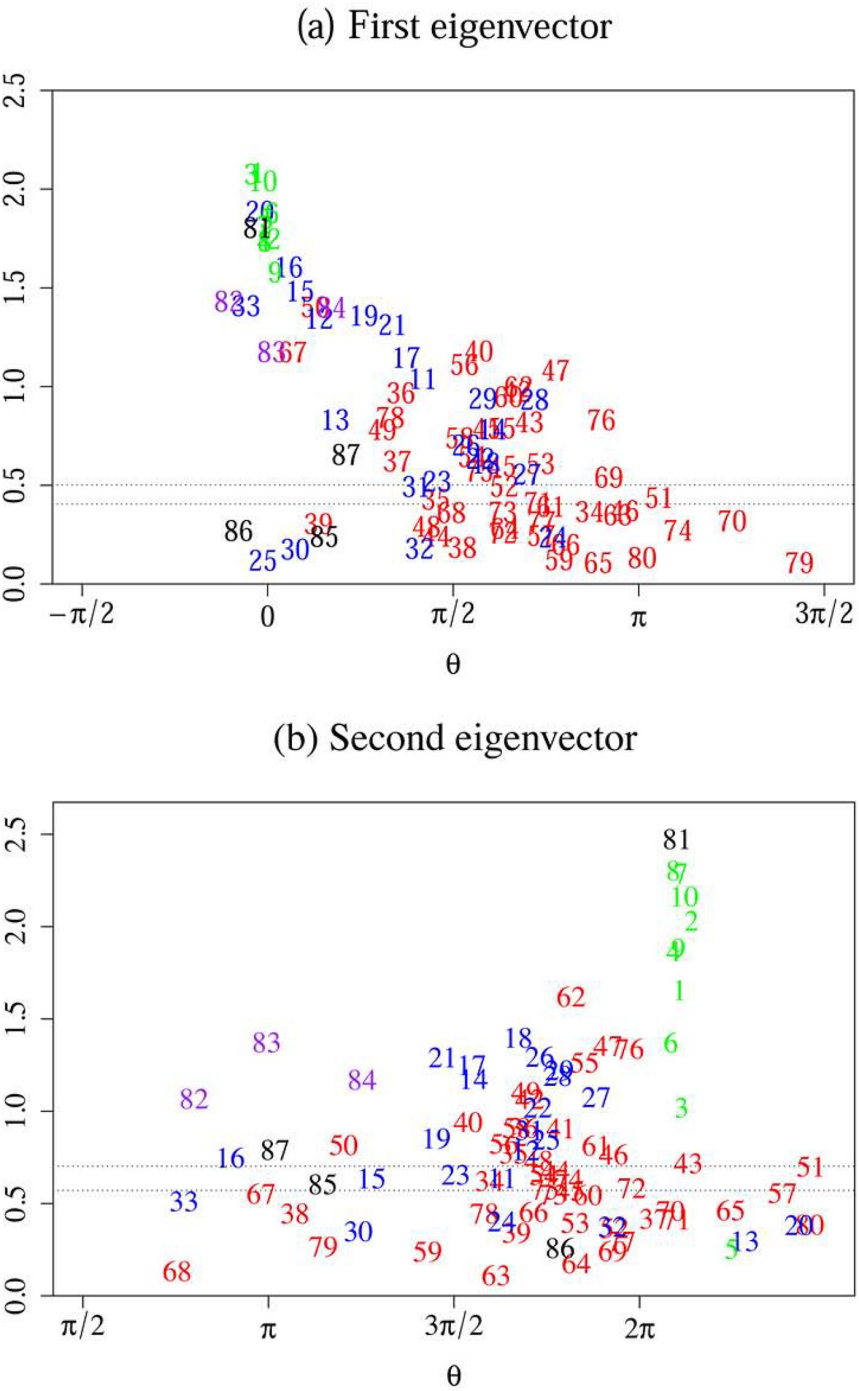
The eigenvectors associated with the largest and second largest eigenvalues. The upper panel plots the complex components of the first eigenvector in a phase-magnitude plane with dotted lines which are the criteria of the auxiliary random variable method to detect significant components at 5% (lower line) and 1% (upper line) significance levels. The lower panel is the same plot as the upper one, but for those of the second eigenvector. According to the present definition of the Fourier transformation, a price changes ahead of (behind) prices on its right-hand (left-hand) side.

Fig 3 shows the complex components of the first and second eigenvectors. Note that the significant eigenvectors generate the dynamics of the entire group of mesoprices, and thereby determine the aggregate price index. The components of these eigenvectors enable us to understand the missing link between the mesoprices and the aggregate price, and also the nature of the driving force behind the macroeconomic variables.

In Fig 3, the vertical axis indicates the absolute value whereas the horizontal axis reflects the phases. The absolute value of each component in a significant eigenvector measures the extent to which the corresponding price (#1–80) or macro-variable (#81–87) contributes to the eigenmode, namely it indicates significant movements in the prices as a whole. On the other hand, the phase difference between a pair of components in a significant eigenvector represents lead-lag relationships between the corresponding pair of prices and macroeconomic variables in the eigenmode. Thus, the information presented in Fig 3 summarizes the dynamics of mesoprices, which generates changes in the aggregate price. The number shown in this figure is intended to identify each of the prices (#1–80) and macroeconomic variables (#81–87).

Prices of which the components have a large magnitude in the eigenvectors play an important role in their correlation structures. To determine whether prices and macroeconomic variables are statistically significant components in the eigenvectors, we reiterated the CHPCA for the price data by adding an auxiliary random time series as the 88th component. The auxiliary component can have a finite magnitude in the eigenvectors of the CHPCA. This is merely an outcome of random fluctuations, such that only those components of the eigenvectors of which the magnitude is larger than that of the auxiliary component should be taken into consideration. We then determined the 5% and 1% significance levels as regards the magnitude of the eigenvector components by collecting 10,000 samples with different random time series. Although the basic structures of the two eigenvectors are robust against the addition of such a random time series, not all of the components are statistically significant. In Fig 3, the two horizontal dotted lines indicate the 1% (upper line) and 5% (lower line) significance levels, respectively. This approach enables us to dismiss components with an absolute value below the 1% significance level as insignificant. Tables 4 and 5 list the significant components at the 1% level for the 1st and 2nd eigenvectors, respectively.

**Table 4:** The first eigenvector.

Abs	*θ* [rad]	Items
1.43	0	82 Index of Business Condition Leading Index
1.41	0.15	33 PPI Scrap & waste
2.07	0.19	3 IPI Metals & related products
2.08	0.25	1 IPI Foodstuffs & feedstuffs
1.80	0.25	81 US Dollar to Japanese Yen Exchange Rate
1.89	0.26	20 PPI Nonferrous metals
2.04	0.29	10 IPI Other primary products & manufactured goods
1.74	0.29	4 IPI Wood, lumber & related products
1.81	0.30	7 IPI General purpose, production & business oriented machinery
1.73	0.3	8 IPI Electric & electronic products
1.85	0.32	5 IPI Petroleum, coal & natural gas
1.17	0.36	83 Index of Business Condition Coincident Index
1.88	0.37	6 IPI Chemicals & related products
1.75	0.38	2 IPI Textiles
1.58	0.39	9 IPI Transportation equipment
1.60	0.51	16 PPI Petroleum & coal products
1.17	0.54	67 CPI Private transportation
1.48	0.60	15 PPI Chemicals & related products
1.40	0.73	50 CPI Other fuel & light
1.34	0.76	12 PPI Textile products
1.40	0.87	84 Index of Business Condition Lagging Index
0.83	0.90	13 PPI Lumber & wood products
0.65	0.99	87 Contractual cash earnings (Manufacturing)
1.36	1.14	19 PPI Iron & steel
0.78	1.30	49 CPI Gas
0.84	1.37	78 CPI Personal effects
1.31	1.39	21 PPI Metal products
0.62	1.43	37 CPI Dairy products & eggs
0.97	1.46	36 CPI Meats
1.15	1.50	17 PPI Plastic products
1.04	1.64	11 PPI Food, beverages, tobacco & feedstuffs
0.52	1.76	23 PPI Production machinery
0.74	1.95	58 CPI Clothes
1.11	2.00	56 CPI Domestic non-durable goods
0.70	2.01	26 PPI Electrical machinery & equipment
0.64	2.06	54 CPI Bedding
1.18	2.12	40 CPI Oils, fats & seasonings
0.57	2.12	75 CPI Recreational services
0.63	2.13	22 PPI General purpose machinery
0.94	2.15	29 PPI Other manufacturing industry products
0.61	2.17	18 PPI Ceramic, stone & clay products
0.79	2.19	41 CPI Cakes & candies
0.78	2.23	14 PPI Pulp, paper & related products
0.79	2.31	55 CPI Domestic utensils
0.60	2.31	45 CPI Meals outside the home
0.95	2.37	60 CPI Footwear
0.97	2.44	42 CPI Cooked food
1.00	2.45	62 CPI Services related to clothing
0.55	2.53	27 PPI Information & communications equipment
0.82	2.54	43 CPI Beverages
0.93	2.59	28 PPI Transportation equipment
0.61	2.64	53 CPI Interior furnishings
1.08	2.77	47 CPI Repairs & maintenance
0.83	3.15	76 CPI Personal care services
0.54	3.22	69 CPI School fees

The components of the 1st eigenvector with an absolute value greater than 0.502 (1% significance level) are listed in the ascending order of *θ* which is the phase measured with reference to that of Leading Index.

**Table 5:** The second eigenvector.

Abs	*θ* [rad]	Items
1.07	0	82 Index of Business Condition Leading Index
0.75	0.31	16 PPI Petroleum & coal products
0.55	0.57	67 CPI Private transportation
1.37	0.61	83 Index of Business Condition Coincident Index
0.79	0.69	87 Contractual cash earnings (Manufacturing)
0.82	1.27	50 CPI Other fuel & light
1.17	1.42	84 Index of Business Condition Lagging Index
0.85	2.05	19 PPI Iron & steel
1.29	2.11	21 PPI Metal products
0.94	2.32	40 CPI Oils, fats & seasonings
1.25	2.35	17 PPI Plastic products
1.17	2.37	14 PPI Pulp, paper & related products
0.82	2.63	56 CPI Domestic non-durable goods
0.77	2.71	35 CPI Fish & seafood
0.34	2.73	39 CPI Fruits
0.91	2.73	58 CPI Clothes
1.40	2.74	18 PPI Ceramic, stone & clay products
0.91	2.79	36 CPI Meats
0.79	2.81	12 PPI Textile products
1.10	2.81	49 CPI Gas
0.90	2.84	31 PPI Minerals
1.07	2.84	42 CPI Cooked food
1.02	2.91	22 PPI General purpose machinery
0.73	2.92	48 CPI Electricity
1.29	2.93	26 PPI Electrical machinery & equipment
0.85	2.98	25 PPI Electronic components & devices
1.19	3.08	28 PPI Transportation equipment
1.22	3.09	29 PPI Other manufacturing industry products
0.91	3.11	41 CPI Cakes & candies
1.62	3.19	62 CPI Services related to clothing
1.26	3.31	55 CPI Domestic utensils
1.08	3.41	27 PPI Information & communications equipment
0.81	3.41	61 CPI Other clothing
1.35	3.51	47 CPI Repairs & maintenance
0.76	3.55	46 CPI Rent
1.34	3.69	76 CPI Personal care services
1.37	4.04	6 IPI Chemicals & related products
2.30	4.06	8 IPI Electric & electronic products
1.87	4.06	4 IPI Wood, lumber & related products
2.47	4.09	81 US Dollar to Japanese Yen Exchange Rate
1.88	4.10	9 IPI Transportation equipment
1.66	4.11	1 IPI Foodstuffs & feedstuffs
2.29	4.12	7 IPI General purpose, production & business oriented machinery
1.02	4.13	3 IPI Metals & related products
2.16	4.15	10 IPI Other primary products & manufactured goods
0.72	4.19	43 CPI Beverages
2.03	4.21	2 IPI Textiles

All the components of the 2nd eigenvector with an absolute value greater than 0.702 (1% significance level) are listed in the ascending order of *θ* which is the phase measured with reference to that of Leading Index.

The phase difference between prices does not straightforwardly translate into lead-lag relations in real time because the phase of the complex correlation coefficient is a nonlinear average over the phase (and thus time) difference of the Fourier components of the prices. In our particular case, however, we have the business cycle indicators which have well-established lead-lag relations in real time. That is, the leading index leads the coincident index by a few months on average, and the coincident index, in turn, leads the lagging index by approximately six months [23]. The difference between the leading and lagging indices is roughly *π*/3 to *π*/2 in phase while it is roughly ten months in real time. Given this information, we can infer that the phase difference of *π* between components in the eigenvectors roughly corresponds to 2 years in real time.

In the first eigenmode, the exchange rate (#81) is by far the most dominant macroeconomic variable responsible for leading prices (Fig 3(a)). This is reflected by the business cycle indicators: the leading (#82), the coincident (#83), and the lagging (#84) indices move in conjunction with the exchange rate. In addition, the prices of raw materials and energy sources such as scrap & waste (#33), nonferrous metals (#20), petroleum & coal (#16), and other fuel & light (#50) are synchronized with the exchange rate. Changes in the remaining PPI prices are delayed, and are finally followed by the CPI prices. In short, the exchange rate first affects import prices (#1–10), then producer prices (#11–33), and finally consumer prices (#34–80). It should also be noted that the absolute values tend to decrease from upstream (IPI) to downstream, (PPI and CPI). In fact, the absolute values of many consumer prices are insignificant. External macroeconomic shocks such as changes in the exchange rate and the oil price gradually attenuate in the course of their propagation from upstream to downstream across domestic prices.

The second eigenmode, on the other hand, represents the domestic business condition (Fig 3(b)). It drives domestic prices. The propagation of shocks across prices does not have the same damping behavior as observed for the first eigenmode.

The exchange rate and import prices, except for the price of petroleum, coal & natural gas (#5), also have large absolute values in the second eigenvector, but curiously, they lag behind other variables. In fact, they lie outside of 2*π*. This apparent lag of the exchange rate behind domestic prices is, in fact, nothing but a mathematical necessity, and therefore, we can disregard it as such. This is explained in Appendix B in S1 File.

In both eigenmodes, the nominal wage (#87) plays a notable role in determining the dynamics of domestic prices. It leads the changes in most of the prices. In fact, in Japan, an unprecedented decline in nominal wages contributed to deflation. In contrast to wages, neither money stock (#85) nor the monetary base (#86) is significant in both of the two eigenmodes.

### Robust co-movement of domestic prices

**Fig 4:**
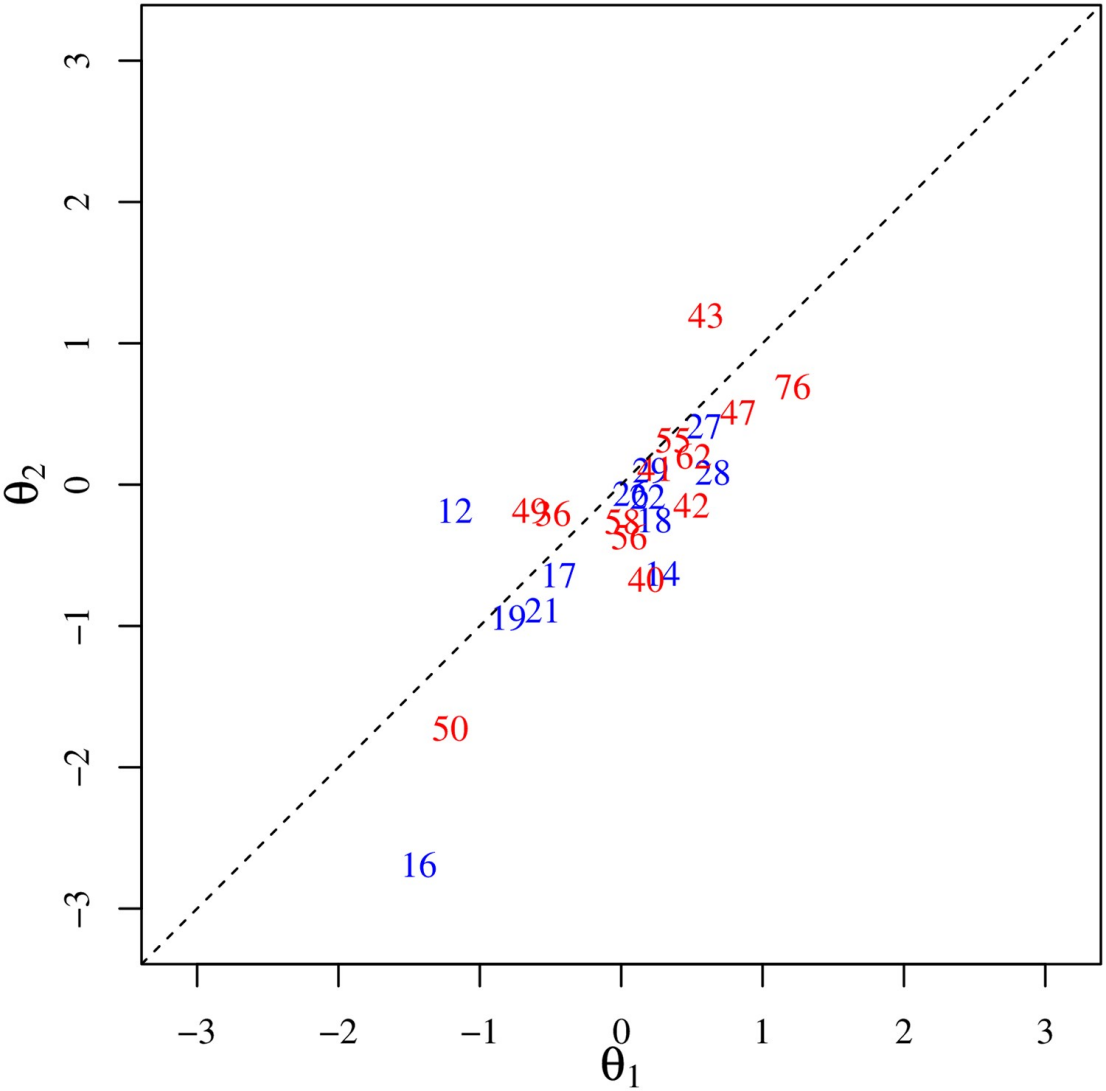
Lead-lag relationship among domestic prices in the first and second eigenmodes. The lead-lag relationship among the significant domestic prices of the first eigenmode is compared with that of the second eigenmode. The phase *θ*_1_ of each price in the first eigenvector is plotted on the horizontal axis and the phase *θ*_2_ of the same price in the second eigenvector, on the vertical axis.

On closer inspection, the first and second eigenvectors reveal that the lead-lag relationships among domestic prices, namely PPI and CPI, in the two eigenmodes are, in fact, quite similar to each other. Fig 4 compares the phases of the significant domestic prices in the first eigenvector with the corresponding phases in the second eigenvector and shows that the prices are well aligned on the correlation plot. We can also confirm the strong resemblance between the lead-lag relations of domestic prices in the two eigenmodes by taking the inner product of the corresponding complex vectors. The absolute value of this inner product was determined to be 0.73, which is highly significant; the associated *p* value is extremely small, i.e., 1.5 × 10^−23^.

This means that domestic prices are characterized by robust internal dynamics irrespective of the forces driving these prices, that is, the exchange rate accompanied by import prices in the first eigenmode or the domestic business climate in the second eigenmode. Domestic prices are thereby interconnected via their mutual interactions to form a network of correlation. The robustness of this property is further confirmed in Appendix C in S1 File, in which we present the results of the analysis we carried out by dividing the data into two periods.

**Fig 5:**
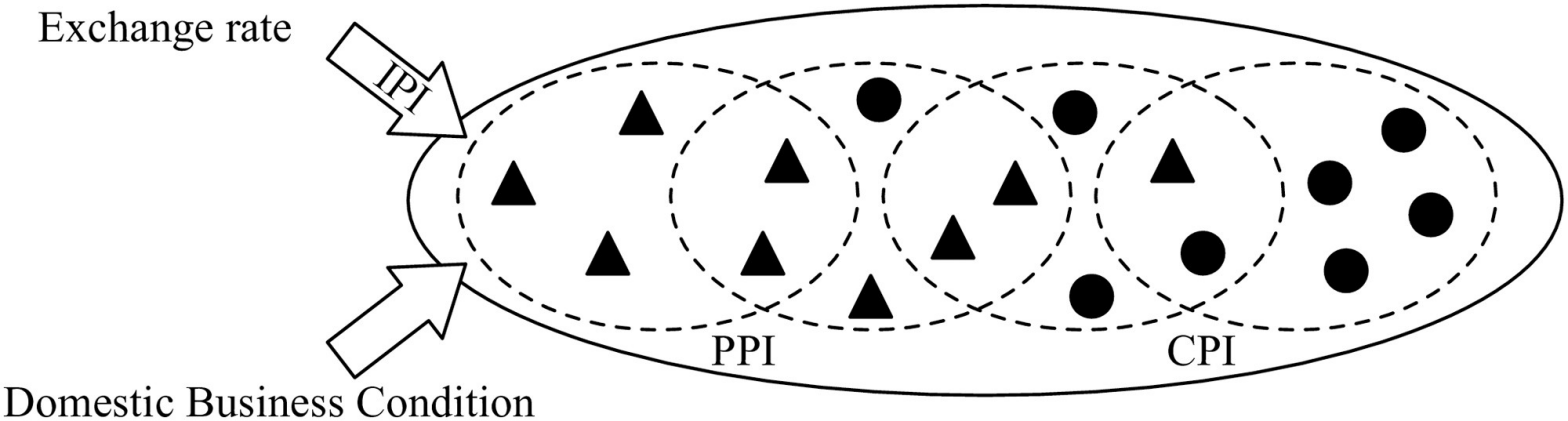
Dynamics of individual prices in clusters. Schematic diagram of comovement of domestic prices originating from their mutual interactions with its driving forces, the dollar-yen exchange rate in the first eigenmode and domestic demand in the second eigenmode. The filled triangles and circles represent individual prices belonging to the PPI and CPI categories, respectively.

The important point is that this network of correlation arises *by way of clusters of individual prices* as schematically depicted in Fig 5. In the next section, we explicitly demonstrate the presence of these clusters of domestic prices (CPI and PPI).

## Price clusters

Most theoretical models routinely assume that firms face a common distribution of idiosyncratic shocks to the relevant variable such as adjustment costs and demand for their products. Firms are therefore assumed to be symmetric. However, with respect to changing prices, firms are not symmetric; instead, they form clusters. In this section we identify these clusters resulting from changes in the domestic prices. We accomplish this by resorting to new methods known as the Hodge decomposition and Percolation analysis.

### Hodge decomposition

Information from the two significant eigenvectors is summarized in the form of the significant correlation matrix. In general, the correlation matrix in (3) can be written as follows:

$$\begin{array}{c} \tilde{C}=\sum_{n=1}^{N}\lambda^{\left(n\right)}V^{\left(n\right)}V^{(n)†}. \end{array}$$ (4)

The significant correlation matrix is defined by limiting the sum to the significant eigenmodes, namely two in the present case.

$$\begin{array}{c} {\tilde{C}}^{\left(\text{sig}\right)}=\sum_{n=1}^{2}\lambda^{\left(n\right)}V^{\left(n\right)}V^{(n)†}. \end{array}$$ (5)

Its phase *θ*_*αβ*_ in ${\tilde{C}}_{\alpha \beta }^{\left(\text{sig}\right)}=\left|{\tilde{C}}_{\alpha \beta }^{\left(\text{sig}\right)}\right|\;e^{i\theta_{\alpha \beta }}$ represents the time-delay of the price *α* relative to the price *β*. Then the question arises as to how to identify the leading prices and to arrange them in the order from upstream (leading sectors) to downstream (lagging sectors), which might be difficult.

For example, suppose that price #1 leads price #2 (*θ*_12_ > 0), price #2 leads price #3 (*θ*_23_ < 0), and price #3 leads price #1 (*θ*_31_ < 0). In this case, we would need to find a way in which to extract the upstream-downstream structure among prices #1, 2, and 3.

The Hodge decomposition would be able to solve this problem. The first step is to construct a network of mesoprices, where nodes *α* = 1, 2, ⋯, *N* are the mesoprices and the links (edges) are the phases *θ*_*αβ*_. Because the absolute value $|{\tilde{C}}_{\alpha \beta }^{\left(\text{sig}\right)}|$ is the strength of the correlation between price *α* and price *β*, we limit the links only to those pairs of prices of which the strength of correlation is above a specified threshold,

$$\begin{array}{c} |{\tilde{C}}_{\alpha \beta }^{\left(\text{sig}\right)}|>C^{\left(\text{th}\right)}. \end{array}$$ (6)

Although we should be able to choose a value for this threshold *C*^(th)^ that is sufficiently large to enable insignificant correlations to be dropped, if we were to choose an excessively large value, the resulting network would obviously become disconnected (in the weak sense). Therefore, we choose this threshold as large as possible under the restriction that the network is weakly connected.

We may regard the value of the link *θ*_*αβ*_ as a “flow” from the price *β* to the price *α* because we seek to identify the upstream-downstream structure in this dataset. The Hodge decomposition divides this flow into two parts:

$$\begin{array}{c} \theta_{\alpha \beta }=\theta_{\alpha \beta }^{\left(c\right)}+\theta_{\alpha \beta }^{\left(g\right)}. \end{array}$$ (7)

The first term on the right-hand side is known as “a circular flow”, which satisfies a conservation property,

$$\begin{array}{c} \sum_{\beta =1}^{N}\theta_{\alpha \beta }^{\left(c\right)}=0. \end{array}$$ (8)

The second term is a gradient flow, which can be written in the following form:

$$\begin{array}{c} \theta_{\alpha \beta }^{\left(g\right)}=\phi_{\beta }-\phi_{\alpha }. \end{array}$$ (9)

This yields the “Hodge potential” *ϕ*_*α*_ for *α* = 1, ⋯, *N*, which we use as a measure of the upstream-downstream structure. In fact, in the absence of circular flow, the order of the prices determined by their Hodge potential values is equivalent to the lead/lag structure indicated by the phases *θ*_*αβ*_. When this phase is small, the difference in the Hodge potential values is small. In this sense, the Hodge decomposition is a natural extension of the phases of the eigenvector components shown in Fig 3, taking into account both of the phases in eigenvector #1 and #2. A detailed explanation of the Hodge decomposition is provided as Supporting Information in Appendix D in S1 File.

**Fig 6:**
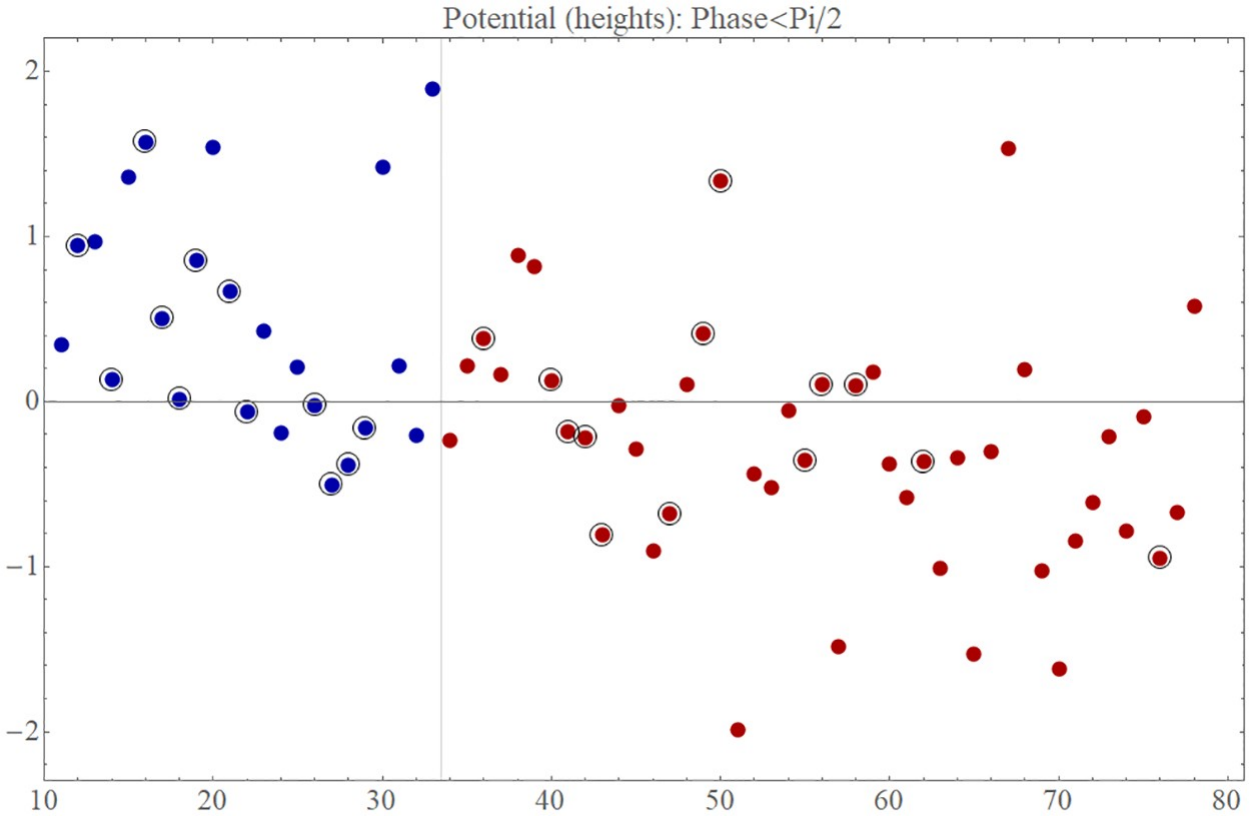
Hodge potentials of the PPI and CPI prices.

The values of the Hodge potentials thus determined are plotted in Fig 6. We readily observe that the Hodge potential of prices in the PPI categories (ID = 11–33) tends to be higher, which means that they tend to be upstream, with few exceptions.

**Fig 7:**
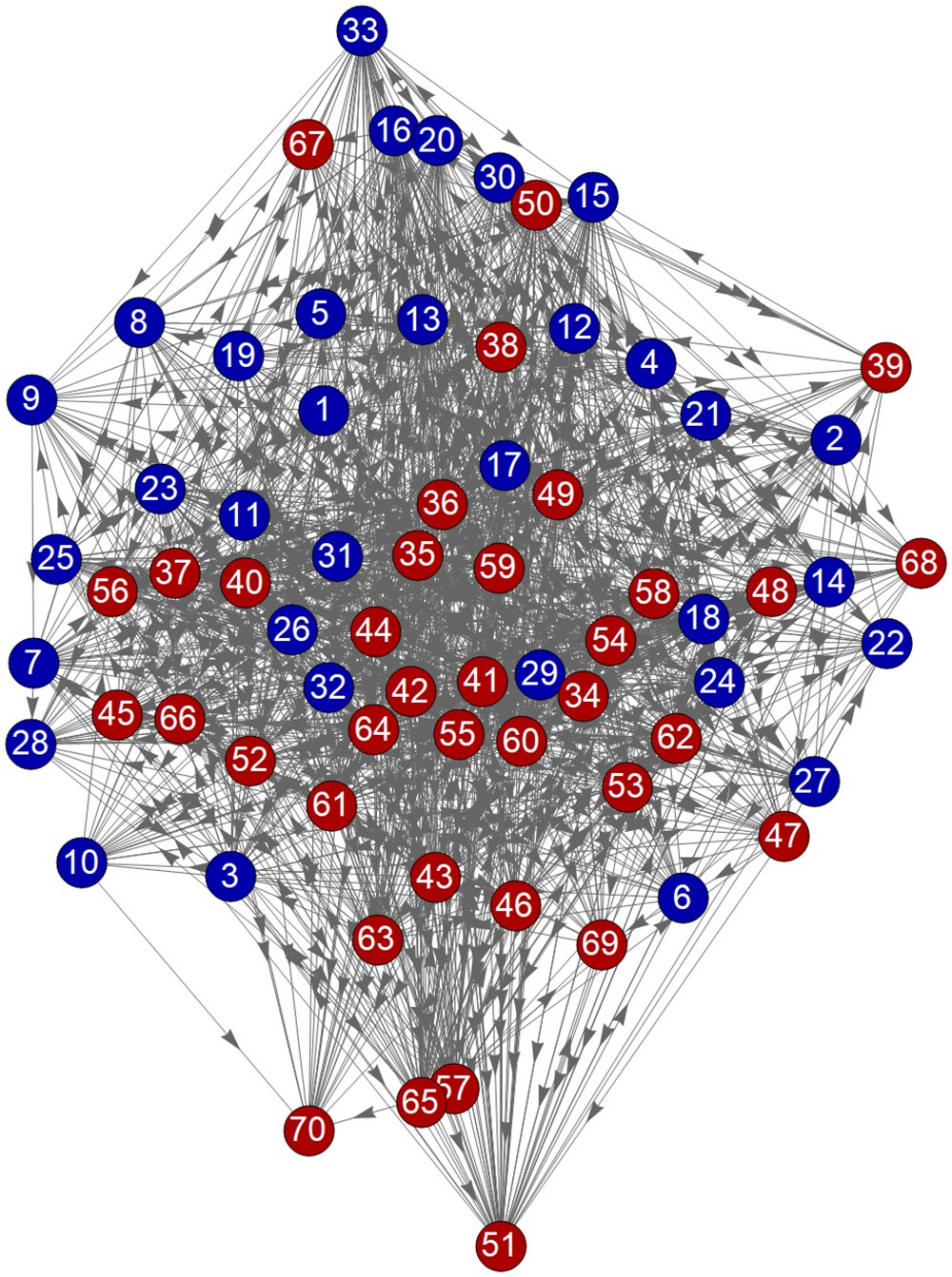
Synchronization network.

The Hodge potentials enable us to visualize the network of prices by using information on the correlation with time lead/lag, which we refer to as the “Synchronization Network.” In Fig 7, the vertical coordinate is the Hodge potential and the value of the horizontal coordinate of each node (price) is fixed by optimization using the Charge-Spring method with the link information.

**Fig 8:**
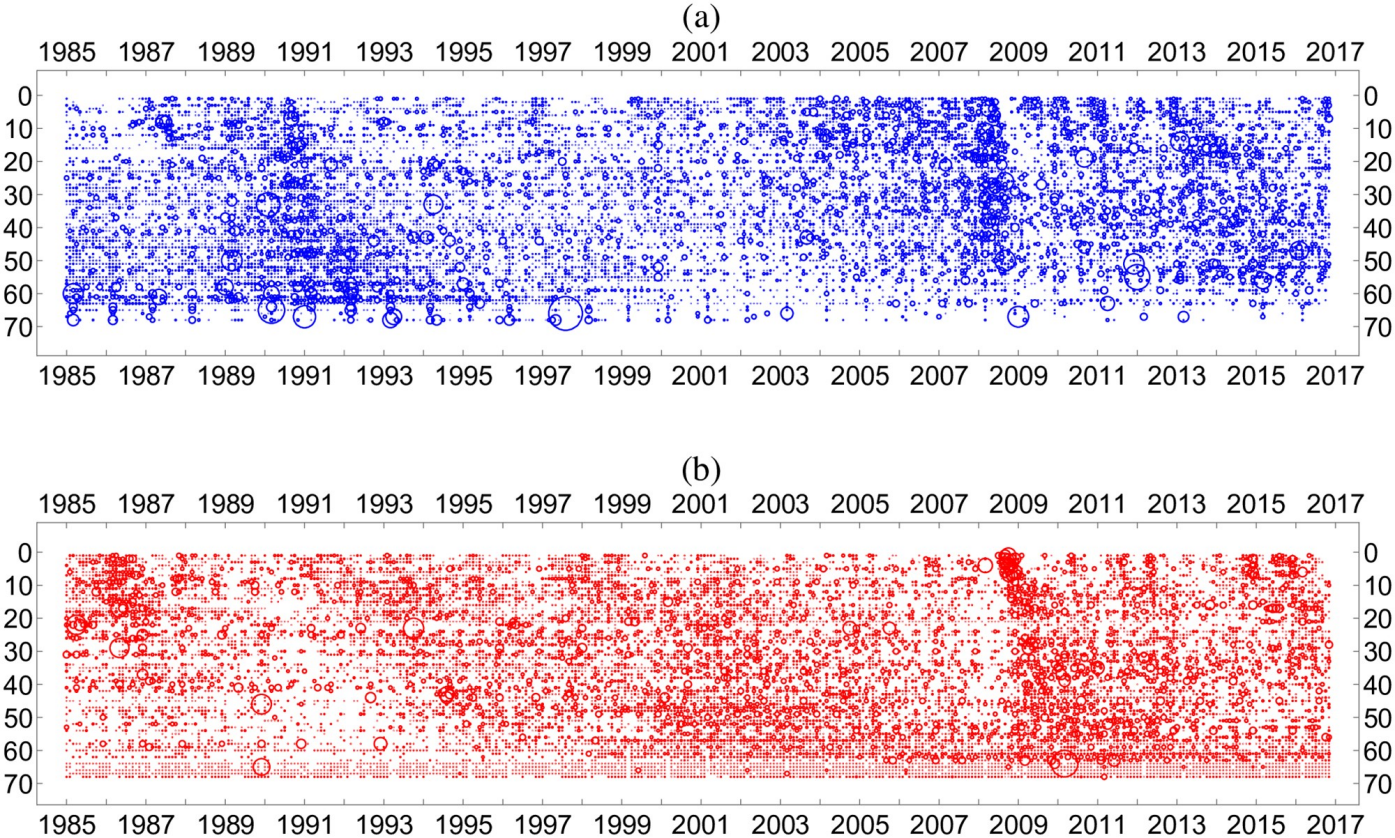
Temporal changes of individual prices. Standardized monthly log differences of individual prices and macroeconomic variables. The domestic price data (PPI and CPI) are exclusively plotted here, and the positive and negative changes are depicted by circles in the panels (a) and (b), respectively. The variables are ordered by their Hodge potential values.

The fact that the Hodge potential determines the order in which changes occur can be demonstrated by plotting the data in the order of the Hodge potential as in Fig 8. Here, each row shows the change in the time series of a price. These rows of prices are ordered vertically, not by their original identification numbers (1-87), but in descending order of the values of their Hodge potentials from top to bottom. We readily observe that changes that occurred in prices in the upper rows (higher in Hodge potential) gradually propagate to those in lower rows, forming clusters that extend from the upper left (high in Hodge potential and earlier in time) to the lower right (low in Hodge potential and later in time). We next present a more refined way to identify these clusters.

### Percolation model

Needless to say, the visual detection of price clusters in Fig 8 is subjective. A more rigorous identification of these price clusters would be possible by using the percolation model [24]. A more rigorous approach is necessary because each cluster comprises a set of price changes that are linked to each other as a result of their “similarity.” Here, the degree of similarity is defined as follows.

**Fig 9:**
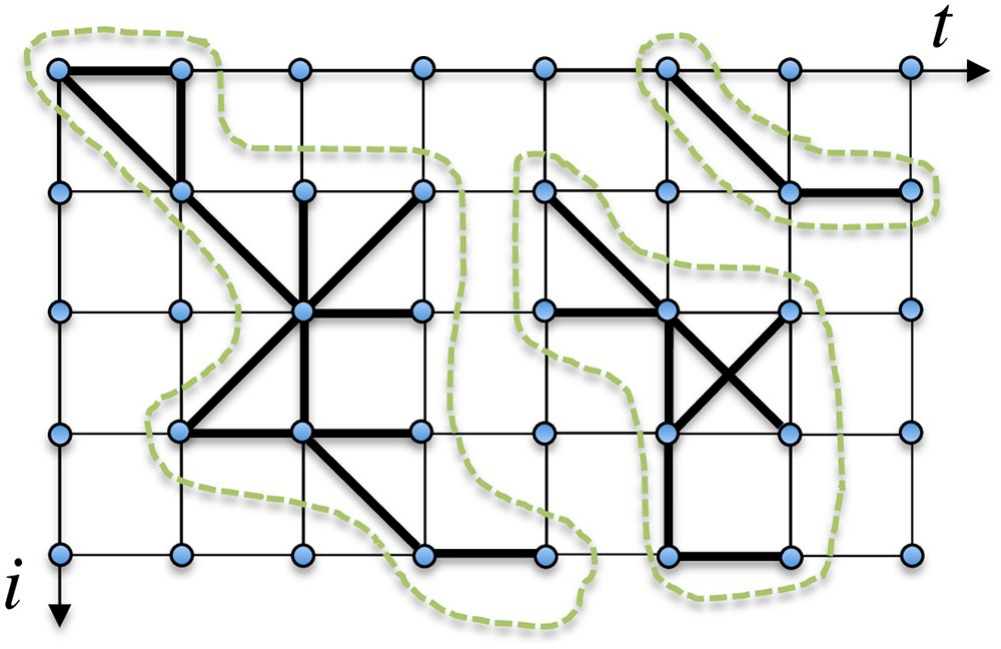
Illustration of the cluster detection. This diagram illustrates the algorithmic way explained in the text for detecting price clusters in Fig 8. Prices are arranged on a square lattice and the thick line between a pair of prices indicates that the two prices are connected according to the criterion (11). Here we find three price clusters, which are encompassed with dotted lines.

First, the data are placed on a square lattice (see Fig 9). The first and second neighbors of each site are obvious candidates for linkage. We measure the strength *g*_*αβ*_ of the coupling between price *α* at time *t* and neighboring price *β* at time *t*′ (*t*′ = *t* or *t*′ = *t* ± 1) by obtaining the geometric mean of their monthly changes *w*_*α*_(*t*) and *w*_*β*_(*t*′):

$$\begin{array}{c} g_{\alpha \beta }\left(t,t^{\prime }\right)=\sqrt{w_{\alpha }\left(t\right)w_{\beta }\left(t^{\prime }\right)}\;. \end{array}$$ (10)

Two neighboring prices are regarded as being *linked* if their coupling constant is larger than a certain threshold *g*_*c*_:

$$\begin{array}{c} g_{\alpha \beta }\left(t,t^{\prime }\right)>g_{c}\;. \end{array}$$ (11)

Obviously, the identification of price clusters depends crucially on the choice of *g*_*c*_. If we were to assign an excessively small value to *g*_*c*_, the prices would fragment to a number of tiny clusters. However, should the value of *g*_*c*_ be overly large, on the other hand, most prices would be connected to form a single cluster. Thus, careful adjustment of *g*_*c*_ close to the percolation threshold in the price lattice system could be expected to lead to the formation of various scales of clusters with a power law distribution. Near the percolation threshold, we can thereby extract information on the clustering properties of prices. This algorithm for detecting price clusters is illustrated in Fig 9.

We iteratively performed the percolation calculations with varied *g*_*c*_ and found the percolation transition to take place in the vicinity of *g*_*c*_ = 0.55 in the model system for both positive and negative changes in the prices. Incidentally, the total number of clusters obtained for *g*_*c*_ = 0.55 are 789 and 693 for positive and negative changes in the prices, respectively. In Supporting Information Appendix E in S1 File, we present the results for cluster detection with different *g*_*c*_ values that correspond to supercritical and subcritical conditions. It becomes clear that the adjustment of *g*_*c*_ to the percolation transition is effective for identifying meaningful clusters.

In passing, we note that the price clusters observed in Fig 8 are prolonged from top to bottom to form a stripe-like structure and are far from being a collection of spheres. This means that the k-means method, a standard clustering method that is often used, may have encountered a problem when attempting to detect these clusters, considering their prolonged shape. On the other hand, the flexibility of the present clustering method based on the percolation model is such that clusters of any shape are detectable.

**Fig 10:**
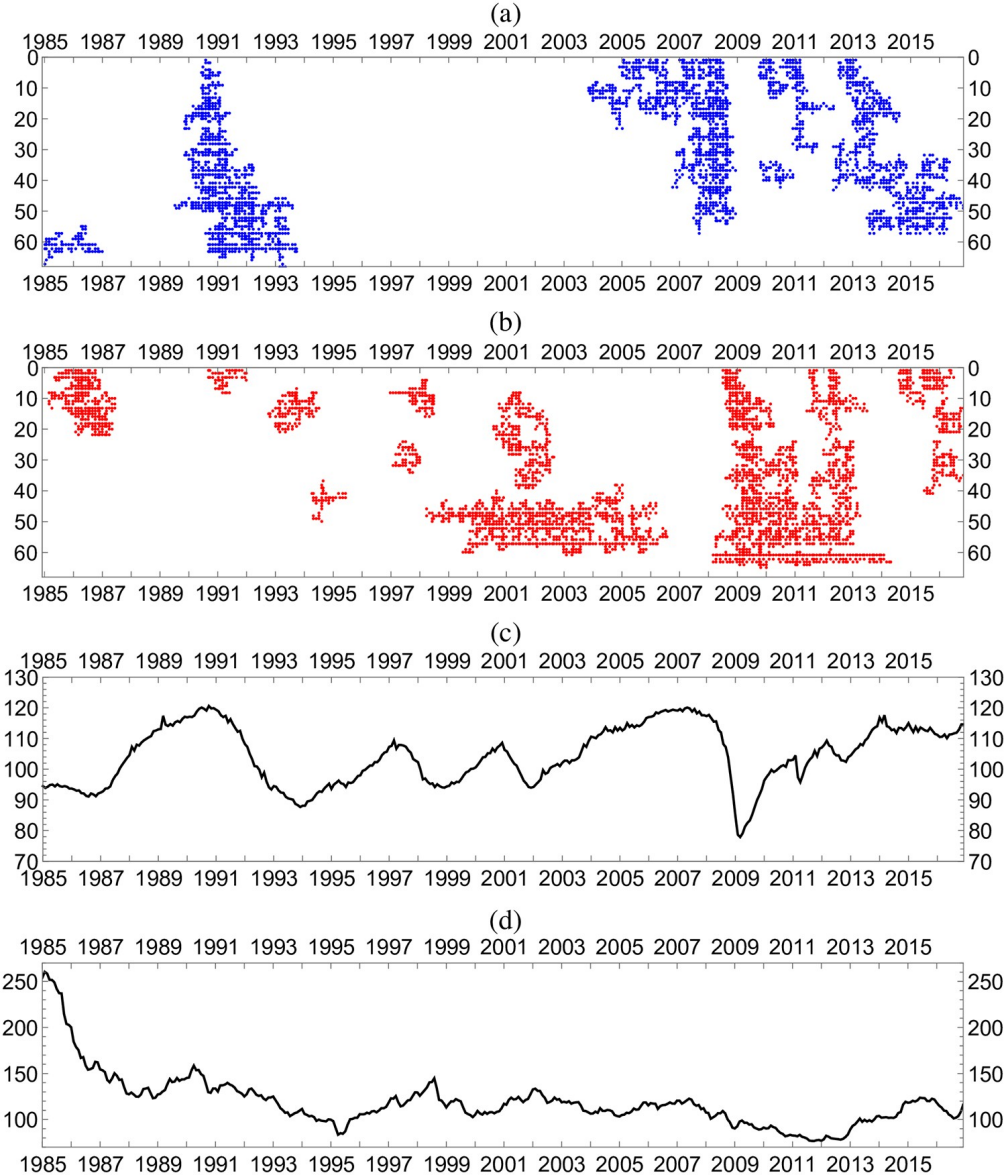
Price clusters with macroeconomic variables. The panels (a) and (b) show the 20 largest clusters as detected by the percolation analysis with *g*_*c*_ = 0.55 for positive and negative changes of prices in Fig 8(a) and 8(b). The panels (c) and (d) show temporal variations of the coincident index and the dollar-yen exchange rate for comparison of their behavior with formation of the price clusters.

Fig 10 shows the major price clusters identified above. We focus on two periods in which the formation of clusters drastically changed. First is the post-Plaza Agreement period (1985-87), and second, the Great Recession (2008-09).

After the Plaza Agreement in the fall of 1985, the yen started appreciating and strengthened from 250 yen per dollar to 120 yen within 2 years (see Fig 10(d)). This sharp appreciation of the yen prompted a decline in import prices and related producer prices, but not in consumer prices (Fig 10(b)). This corresponds to the first eigenmode in the analysis presented in the previous section. Noteworthy is that this period is characterized by the absence of a cluster of price increases.

Beginning in 1987, Japan experienced a major boom sustained by substantial increases in asset prices, which in retrospect, turned out to be bubbles (Fig 10(c)). Prices rose, first in the upstream sectors, and subsequently in downstream sectors. Consequently, in Fig 10(a), the clusters are gradually repositioned from the northwest to the southeast over time. During the period 1987–90, the formation of a cluster of declining prices was not observed. After the price bubble burst and the Japanese economy entered a more severe recession in 1991, upstream prices started declining while downstream prices continued rising.

The year 2008 also provides us with an excellent opportunity for a case study. The long-lasting boom, which started in 2002, eventually generated price increases again from the upstream to the downstream sectors for the period from 2006 to the fall of 2008 (Fig 10(a)), for which no cluster of price decline is identified (Fig 10(b)). In the fall of 2008, Japan’s notorious deflation finally appeared to end. However, the bankruptcy of Lehman Brothers turned the tide. Prices in the different clusters abruptly started declining.

**Fig 11:**
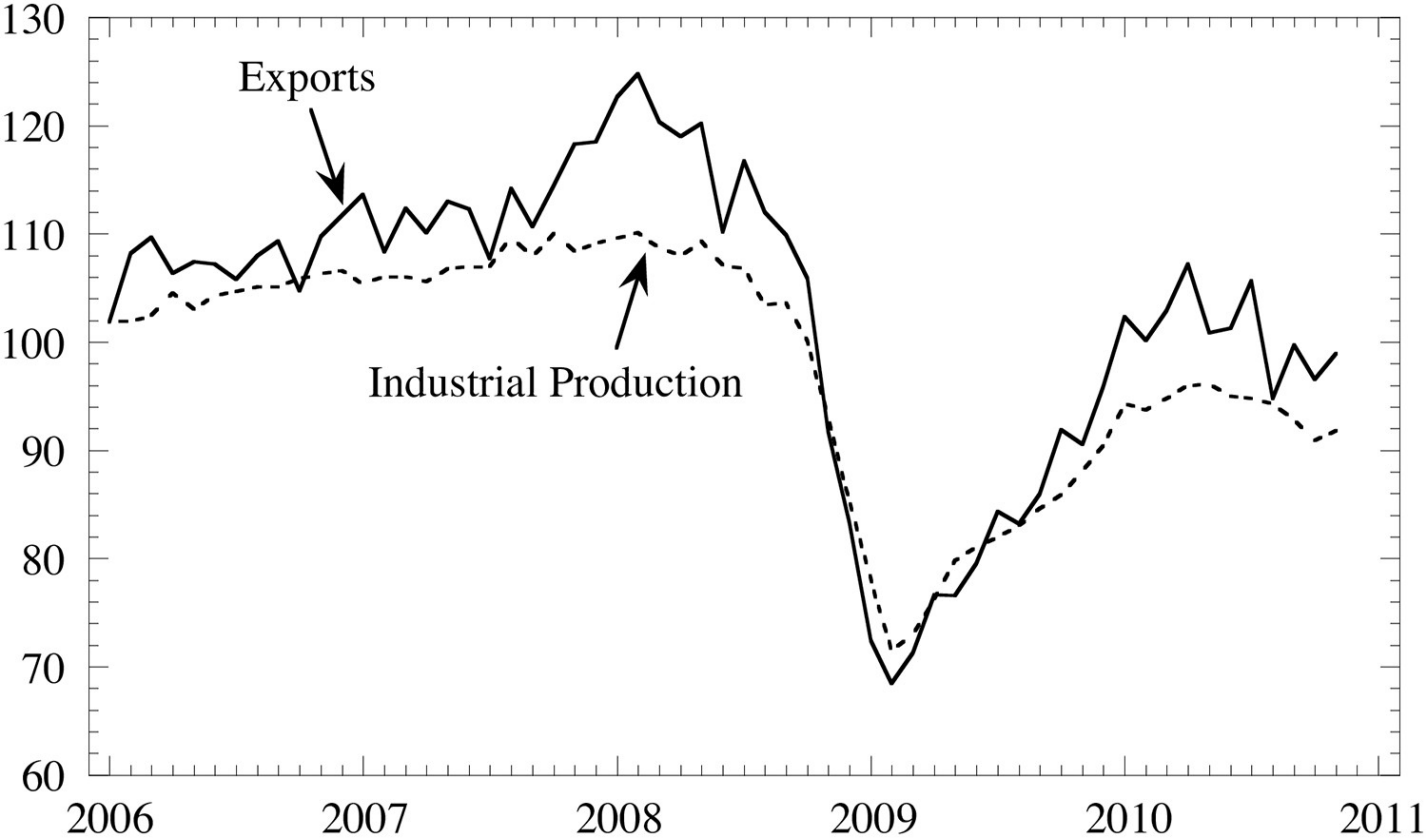
Indices of exports (solid line) and industrial production (dashed line) normalized to 100 at 2005.

It should be noted that, unlike the U.S. economy, Japan’s “Great Recession” was *not* caused by a financial crisis; in fact, the Japanese financial system was basically stable. Japan’s sharp recession in 2009 as shown in Fig 10(c) was caused by an unprecedented decrease in exports due to the global recession: see Fig 11 ([22]).

The fall in the aggregated demand, exports in particular, prompted the Japanese economy into recession, thereby causing prices to decrease (Fig 10(b)). Rising prices abruptly became falling prices. This change due to the weakened domestic economy is captured by the second eigenmode in CHPCA in the previous section.

Beginning in 2013, the yen started depreciating (Fig 10(c)), while the price of oil rose simultaneously. As a result, prices of imported goods rose, and their increases were propagated from upstream prices to downstream prices. Fig 10(a) shows that, once again, clusters were repositioned from the northwest to the southwest during the period.

## Concluding remarks

The world today is in an age of low inflation. Although low inflation may be welcome, it is next only to deflation, which can threaten the macroeconomy. Deflation, even if it is not the collapse in the prices Irvin Fisher faced during the 1930’s, still has a serious affect on monetary policy, particularly when the nominal interest rate is extremely low, say zero. The Bank of Japan, the U.S. Federal Reserve, and the European Central Bank have all faced the zero interest bound amid low inflation or even deflation. Central banks have resorted to sizable quantitative easing (QE), but they continue to experience difficulties in achieving the target rate of inflation, namely 2%.

The fact that prices do not rise remains a puzzle. Obviously we need to know more about price changes.

Although Berger and Vavra [14] showed that the frequency and variance of price changes are countercyclical, their work did not really explore the kind of firms that change their prices. In fact, in most theoretical models, it is routinely assumed that firms face the common distribution of idiosyncratic shocks. In other words, firms are assumed to be symmetric. Our analysis demonstrates that this assumption is not borne out by facts.

We analyzed the interactions of mesoprices by using a new method named Complex Hilbert Principal Component Analysis (CHPCA). Although this method is little known in economics, it has been successfully applied in many fields of the natural sciences. This method considers the lead-lag relationships that are present in mesoprice dynamics. We note that ordinary (real) principal component (factor) analysis fails to uncover hidden common factors when significant leads and lags are present in the variables under investigation. CHPCA enables us to capture the systematic behavior of the group of mesoprices in its entirety, and thereby the aggregate price and the nature of macroshocks driving the changes. We applied CHPCA to 80 mesoprices and 7 macroeconomic variables and found two dominant factors (eigenmodes) to be responsible for generating the systematic dynamics of mesoprices, as a whole, and thereby the aggregate price.

The first significant eigenmode generating the systemic fluctuations of individual prices is significantly correlated with the exchange rate and the price of crude oil. In an open economy such as that of Japan, changes in the exchange rate and oil price affect the prices of imported goods and services without lagging, and they, in turn, change the costs of energy and materials used in the production of a wide range of goods and services. Even though they may lag, many prices follow suit. Gopinath, Itskhoki, and Rigobon [25] found that, in the case of the U.S. exchange rates also systematically affect import prices, but that the elasticity of import prices with respect to changes in the exchange rates is rather small, namely firms adjust prices by only 0.25% for each 1% change in the exchange rate. The case study of the Post-Plaza Agreement period when the yen sharply appreciated from 240 per dollar to 120 amply demonstrates the presence of this mechanism. In fact, in their study of the long-term data (1870–1950) for the U.K. Brown and Ozga [26] found that the most important determinant of British prices was the terms of trade, which was in turn basically determined by the prices of raw materials. It is easy to dismiss this finding by saying that price is nominal whereas the terms of trade are real. However, that is what the data suggest. In terms of the Japanese economy, the real price of energy and the real exchange rate affect the nominal aggregate price.

The second significant eigenmode represents the domestic business climate. In both eigenmodes, nominal wage plays a role in determining the dynamics of domestic prices and leads the change of most prices. In contrast, neither the money stock nor the monetary base is significant.

We found that, irrespective of the nature of the driving forces, domestic prices have robust internal dynamics. Most important is the finding that mesoprices change *in clusters*. The clusters of price changes shift from upstream (raw materials) to downstream (final consumer goods) as a function of time.

These cluster dynamics most likely arise as a result of the input/output relationships in the production of goods and services. The propagation of price change in the form of clusters suggests that the prices of final goods and services are established on the basis of costs rather than expectations. We recall that cost-based mark-up pricing was once said to be prevalent [13, 27]. Eichenbaum, Jaimovich, and Rebelo [28] used scanner data from a US supermarket chain to show that retail prices, reference prices excluding temporary sales in particular, tend to change with the aim of maintaining the mark up over the marginal cost at its average level. This conclusion is in accordance with the following remark made in [29].

“(Recent research on inflationary expectations is) flawed because it placed the information barriers in the wrong place, in an inability to perceive costless macro information, instead of where the information barriers really exist, at the micro level of costs and supplier-producer relationships. Producers of final goods are unable to perceive cost increases of crude and intermediate materials that may be in the pipeline, and they have no choice but to wait until they receive notification of actual cost changes (with the exception of crude materials like oil where prices are determined in public auction markets). ⋯ A fundamental source of persistence is not just explicit wage contracts as analyzed by Taylor, but also explicit or implicit price contracts between suppliers and producers of final goods. Even without contracts, persistence and inertia are introduced by lags between price changes of crude materials, intermediate goods and final goods. For some goods, e.g. cars or aircraft, there are literally thousands of separate intermediate goods, and most of these are made up of further layers of intermediate goods.”

(in [29], pp.32-33).

Deflation and inflation are macroeconomic phenomena. However, we cannot fully understand them by only exploring macroeconomic data because the behavior of an aggregate price index such as the CPI depends crucially on interactions among mesoprices. The results we obtained strongly suggest that, to improve our understanding of the behavior of an aggregate price, namely, inflation/deflation, we should redirect our research from the analysis of price setting on the assumption of a representative firm to that of interactions of firms/sectors based on input/output linkages or intersectoral production networks. Recent theoretical and empirical investigations on business cycles have resurrected the importance of input-output linkages in production [30]. Our analysis suggests that this holds true not only for quantity but also for price.

## Supporting information

S1 File Appendices A–E to the manuscript. (PDF)
