# Supplementary material for: Interindustry linkages of prices—Analysis of Japan’s deflation
Source: PLoS One. 2020 Feb 13;15(2):e0228026. doi: 10.1371/journal.pone.0228026 (PMC7018021; doi:10.1371/journal.pone.0228026)
Supplement: S1 File — (PDF) [file pone.0228026.s001.pdf]

Supporting Information

Interindustry linkages of prices – Analysis of Japan's deflation

Yuichi Kichikawa[1], Hiroshi Iyetomi[2], Hideaki Aoyama[3,4,5*],
Yoshi Fujiwara[5], and Hiroshi Yoshikawa[4,6]

1 Faculty of Science, Niigata University, Niigata 950-2181, Japan
2 Department of Mathematics, Niigata University, Niigata 950-2181, Japan
3 GSAIS, Kyoto University, Kyoto 8306, Japan
4 RIETI, Tokyo 100-0013, Japan
5 Graduate School of Simulations, University of Hyogo, Kobe 650-0047, Japan
6 Faculty of Economics, Rissho University, Tokyo 141-8602, Japan
* hideaki.aoyama@gmail.com

Appendix A Validity Check of the CHPCA

Here we address how effective is the CHPCA in extracting the comovement behavior of prices with lead-lag relations. The validation analysis of the method is carried out using synthetic multivariate time series into which definite lead-lag relations and noises are incorporated.

We begin with preparing the following time series data with the same numbers of N and T that the price data have:

$$x_i(t) = \sin\left(\frac{2\pi}{P}t + \delta_i\right) + \epsilon z_i(t), \tag{S1}$$

where $i = 1, 2, \cdots, N$ and $t = 0, 1, \cdots, T$. The first term in the right-hand side of Eq. (S1) represents a sinusoidal signal of period P with a given phase δ_i, which is randomly chosen in the range $(-\pi/2, \pi/2)$. The second term plays a role of random noises disturbing the signal; ϵ determines the relative strength of noises as compared with the signal and $z_i(t)$ is generated randomly according to the standardized normal distribution. We then apply the CHPCA to the test time series by taking difference for each of them:

$$\Delta x_i(t) = x_i(t) - x_i(t-1). \tag{S2}$$

Figure S1 exemplifies the time series synthesized at three values of ϵ together with their difference; we set $m = 5$ and hence $P = 76.6$ throughout this analysis. The difference of the test data is almost indistinguishable from that of completely random noises at $\epsilon = 0.3$. This is because the intensity of the signal is two orders of magnitude smaller than that of noises. The signal-to-noise ratio S/N in $\Delta x_i(t)$ is approximately given by

$$S/N = \frac{P_{\mathrm{S}}}{P_{\mathrm{N}}} = \frac{1}{4\epsilon^2}\left(\frac{2\pi}{P}\right)^2, \tag{S3}$$

where P_{S} and P_{N} are the signal power and the noise power, respectively. This formula is derived by noting that the difference of $x_i(t)$ can be replaced by its derivative in the condition of $P \gg 2\pi$:

$$\Delta x_i(t) \simeq \frac{2\pi}{P}\cos\left(\frac{2\pi}{P}t + \delta_i\right) + \epsilon_i(t) - \epsilon_i(t-1). \tag{S4}$$

Taking ensemble average of Eq. (S4) over noises, we obtain

$$\left\langle \Delta x_i(t)^2 \right\rangle \simeq \left(\frac{2\pi}{P}\right)^2 \cos^2\left(\frac{2\pi}{P}t + \delta_i\right) + 2\epsilon^2. \tag{S5}$$

The time average of Eq. (S5) finally leads to the power of the total fluctuations:

$$\overline{\langle \Delta x_i(t)^2 \rangle} \simeq \frac{1}{2}\left(\frac{2\pi}{P}\right)^2 + 2\epsilon^2, \tag{S6}$$

where the first and second terms in the right-hand side of Eq. (S6) give P_{S} and P_{N}, respectively. The time-averaged power as given in Eq. (S6) should be relevant in the CHPCA, because it entirely depends on *static* (*equal time*) correlations between complexified time series.

In Fig. S2, we show the probability distribution functions of the eigenvalues of the CHPCA applied to the synthesized data for three values of ϵ in the range $0.2 \le \epsilon \le 0.3$. The largest eigenvalue λ_1 at $\epsilon = 0.2$ is clearly isolated from the bulk of the eigenvalues, and it is statistically significant according to the same criterion adopted in the main text; it is larger than the upper limit λ_c of the largest eigenvalue predicted for the corresponding data which are completely random. On the other hand, λ_1 at $\epsilon = 0.3$ is merged into the bulk of the eigenvalues and it is not statistically significant with $\lambda_1 < \lambda_c$. At $\epsilon = 0.25$, $\lambda_1 (\simeq \lambda_c)$ is in a marginal situation.

Figure S3 depicts the first eigenvectors on complex plane obtained at the same values of ϵ as in Fig. S2. Coinciding with the statistical significance of the largest eigenvalue, the eigenvector at $\epsilon = 0.2$ reproduces well the lead/lag relations embedded in the test data while one cannot detect the hidden relations out of the eigenvector for $\epsilon = 0.3$. This result is more clearly visible in Fig. S4, where the phases extracted from the first eigenvector of the CHPCA are compared with the corresponding phases given on synthesizing the data at $\epsilon = 0.2, 0.25$, and 0.3. We note that the CHPCA works for the test data with the S/N just less than 5%.

From the above test on the CHPCA we can infer that the method is quite effective in extracting the comovement behavior of prices with lead-lag relations. And also we confirm that the lead/lag relations among time series data detected from the eigenvectors of the CHPCA are reliable once their corresponding eigenvalues are judged to be statistically significant.

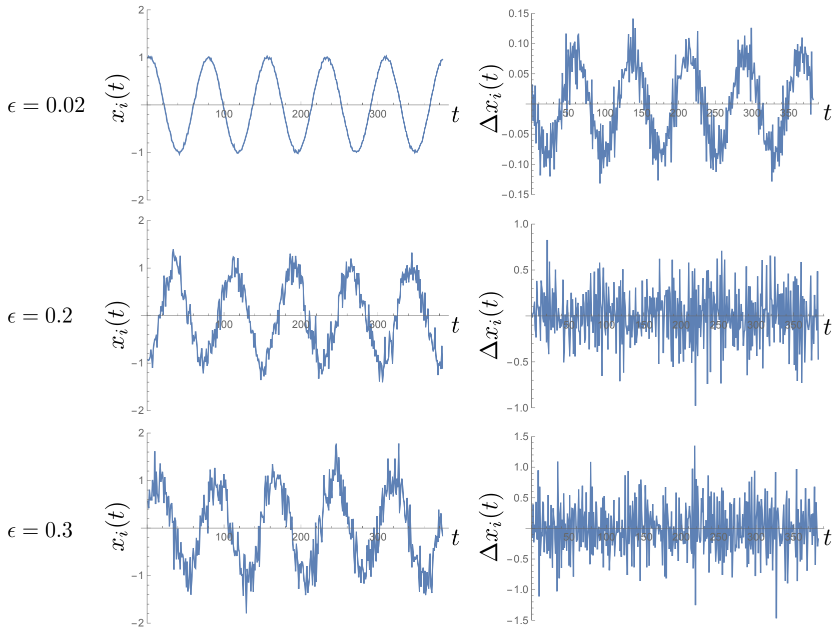

Figure S1: Examples of the synthesized data at three values of ϵ and their difference. The signal-to-noise ratio S/N is 4.2, 0.042, 0.019 for $\epsilon = 0.02, 0.2, 0.3$, respectively.

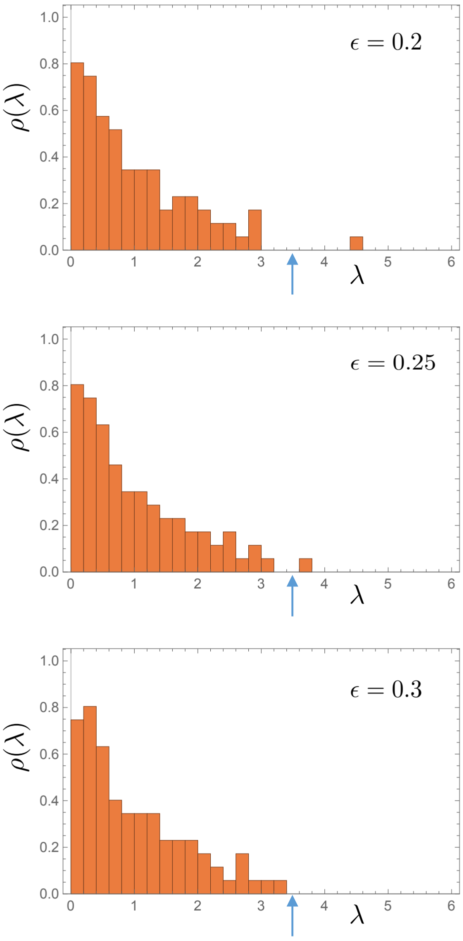

Figure S2: Probability distribution functions of the eigenvalues of the CHPCA applied to the synthesized data for three values of ϵ. The vertical arrow in each panel shows the upper limit of the largest eigenvalue determined at 3σ confidence level for the corresponding data which are completely random.

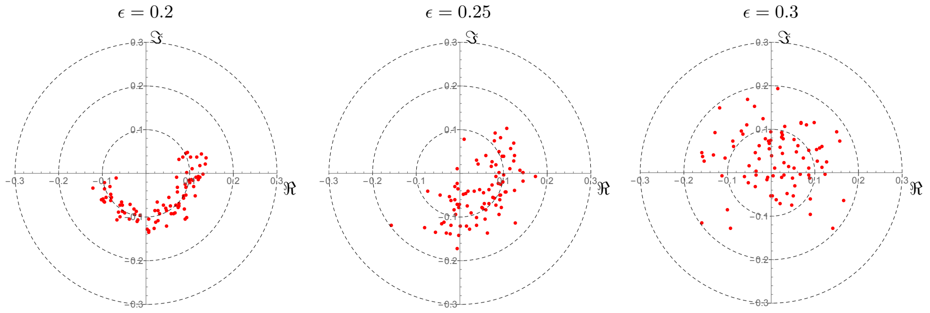

Figure S3: Eigenvectors depicted on complex plane, each of which is associated with the largest eigenvalue in the corresponding panel of Fig. S2.

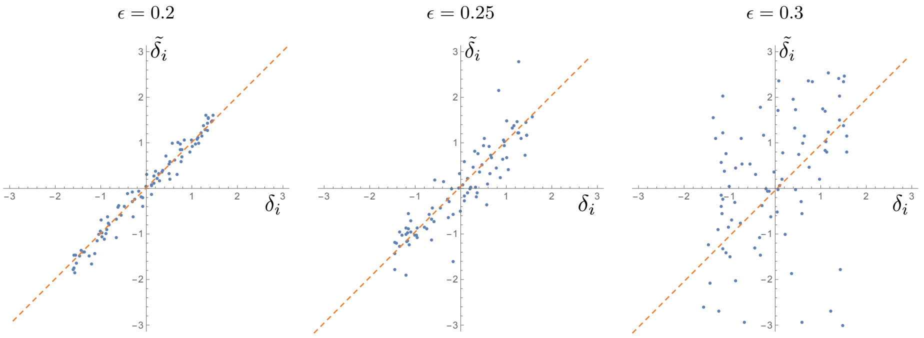

Figure S4: Comparison of given phases of the synthesized data and the corresponding phases extracted from the first eigenvectors of the CHPCA in Fig. S3.

Appendix B Mathematical Structure of Eigenvectors of Two-variable Model

In Section "Dynamics of Prices and Macroeconomic Variables", the exchange rate and import prices, though they have large absolute values, lay behind domestic prices in the second mode (Figure 3(b)). In this appendix, we show that it is nothing but a mathematical necessity in two-variable model. To understand the correlation structures observed in the first and second eigenmodes, we introduce a simple two-variable model. For this purpose, we first replace the group motion of domestic prices by a single collective coordinate, that is, the mode signal of the first eigenmode of the CHPCA applied to the reduced data set in which only domestic pries are retained. Also we replace the dollar-yen exchange rate and import prices by another collective coordinate. Adopting the two collective coordinates reduces the economic system under study to a two-variable model.

In this two-variable model, the complex correlation matrix $\tilde{\boldsymbol{C}}$ has such a reduced form as

$$\tilde{\boldsymbol{C}} = \begin{pmatrix} \sigma_1 & \sigma_{12} \\ \sigma_{12}^* & \sigma_2 \end{pmatrix} , \tag{S7}$$

where σ_1 and σ_2 are the variances of the collective coordinates for domestic prices and the exchange rate accompanied by import prices, respectively, and σ_{12} is a complex correlation coefficient between the two coordinates.

If σ_1 and σ_2 take an identical value σ, the two eigenvalues $\lambda_\pm$ are calculated as

$$\lambda_\pm = \sigma \pm |\sigma_{12}| , \tag{S8}$$

with their eigenvectors $\boldsymbol{V}_\pm$ given by

$$\boldsymbol{V}_+ = \begin{pmatrix} 1 \\ \exp(-i\theta) \end{pmatrix} , \ \boldsymbol{V}_- = \begin{pmatrix} 1 \\ -\exp(-i\theta) \end{pmatrix} , \tag{S9}$$

where θ is the phase angle of σ_{12}. We see that the relationship between the comovement of domestic prices and the exchange rate in $\boldsymbol{V}_+$ is reversed in $\boldsymbol{V}_-$. When $0 < \theta < \pi/2$, for example, the exchange rate leads the comovement of domestic prices with phase difference θ in $\boldsymbol{V}_+$, while the exchange rate follows the comovement of domestic prices with phase difference $\pi - \theta$.

In the actual data, we obtain $\sigma_1 = 7.81$ and $\sigma_2 = 7.42$. The former is the largest eigenvalue of the submatrix of $\tilde{\boldsymbol{C}}$ for domestic prices, and the latter, that for the exchange rate and import prices. Thus, the condition $\sigma_1 = \sigma_2$ is approximately satisfied. The model with (S9) of $\boldsymbol{V}_+$ and $\boldsymbol{V}_-$ therefore well explains the correlation structures in the two dominant eigenmodes. The exchange rate drives the comovement of domestic prices in the first eigenmode to fix the phase difference θ between the two collective coordinates. On the other hand, in the second eigenmode, the lead-lag relationship of the exchange rate with the comovement of domestic prices is automatically determined by $\pi - \theta$. This is basically what we observe in Figure 3(b). It is simply a mathematical necessity in a two-variable model.

Given this mathematical fact, it is not the end of the story. Because replacement of the exchange rate by a completely random time series would result in

the same mathematical relation between $\boldsymbol{V}_+$ and $\boldsymbol{V}_-$ as long as the condition $\sigma_1 \simeq \sigma_2$ is satisfied. The random time series is fixed to the comovement of domestic prices at any phase angle. The remaining issue to be addressed is thereby whether the fixed phase difference θ between the two collective coordinates in the first eigenmode is statistically significant or not. To test statistical significance of the phase angle between the comovement of domestic prices and the exchange rate, we reiterated the CHPCA calculation for the data set in which the exchange rate and import prices are substituted by a random time series with the variance kept the same; the new results serve as a null model. The strength of coupling between the two collective coordinates is represented by the magnitude of σ_{12} and hence by difference of the two dominant eigenvalues as shown in Eq. (S8). Figure S5 demonstrates distribution of $\lambda_1 - \lambda_2$ in the null model. On the other hand, the actual result for $\lambda_1 - \lambda_2$ is 2.401 and its p-value is given as 0.006 according to the null hypothesis. This comparison allows us to infer that the fixed phase angle between the comovement of domestic prices and the exchange rate is statistically meaningful.

In conclusion, the correlation structures in the two dominant eigenmodes are fully understandable with a two-variable model. And also we confirm that the exchange rate is certainly a driving factor for the first eigenmode.

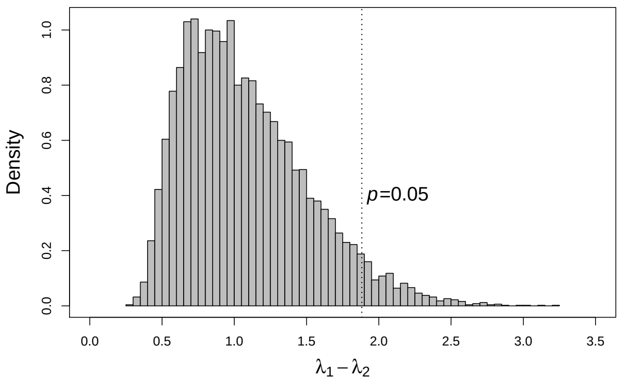

Figure S5: **Distribution of the eigenvalue separation,** $\lambda_1 - \lambda_2$**.** The CHPCA with a random time series in place of the exchange rate and import prices (sampled 10,000 times), where the variance of random time series is kept the same as $\sigma_2 = 7.42$.

Appendix C 2-period analysis

To ascertain the robustness of comovement of domestic prices, furthermore, we prepared two subsets of data out of the whole dataset by separating evenly the period into two parts, January 1985 through December 2000 and January 2001 through December 2016, and applied the CHPCA to each of them. The former half of the whole period begins with the bubble boom and its burst, resulting in the lost decade in Japan. During the latter half, on the other hand, the gradual recovery of Japanese economy was struck by the Lehman crisis and subsequently the Abenomics with quantitative and qualitative monetary easing has appeared to try to make the nation recover from the long-standing recession. We thus see that the two eras are the two subperiods are so distinguishable economically.

Figures S6 and S7 display the eigenvector associated with the largest eigenvalue in the former and latter halves, respectively. Since the IIP's were omitted in these calculations, we have just one mode for comovement of domestic prices in each subperiod. The collective behaviors are astonishingly similar considering that the two eras are featured by totally different economic situations. We can confirm this in a quantitative way by calculating a generalized cosine similarity η between a pair of complex vectors $\boldsymbol{a}$ and $\boldsymbol{b}$, defined as

$$\eta = \frac{|\boldsymbol{a} \cdot \boldsymbol{b}^*|}{\|\boldsymbol{a}\| \, \|\boldsymbol{b}\|} , \qquad \text{(S10)}$$

where $\|\boldsymbol{a}\|$ stands for the norm of $\boldsymbol{a}$. We note that η takes unity for a pair of identical vectors. The generalized cosine similarity for the two eigenvectors shown in Figs S6 and S7 is $\eta = 0.708$. This value is much closer to unity as compared with the result $\eta = 0.372$ obtained at the significance level of 0.05 by randomly shuffling the order of components in the eigenvectors. Also we compare the lead-lag relationship among domestic prices of significant contribution in the two eigenvectors in the same way as in Fig.4. Once again, we observe closeness of the collective behaviors of domestic prices between the well distinguishable eras.

Finally we pay attention to how the macroeconomic variables are related to the collective motion of domestic prices. In both subperiods, the indices of business condition (#82, #83, #84) play a key role in leading the dynamics of domestic prices. In the former half, the monetary variable M2 (#85) is also significant as a leading index for domestic prices. In the latter half, on the other hand, it is replaced by the nominal wage (#87). The change of roles of the two macroeconomic variables may reflect drastic shift of economic conditions over the last 30 years in Japan, while the collective motion of domestic prices remains intact.

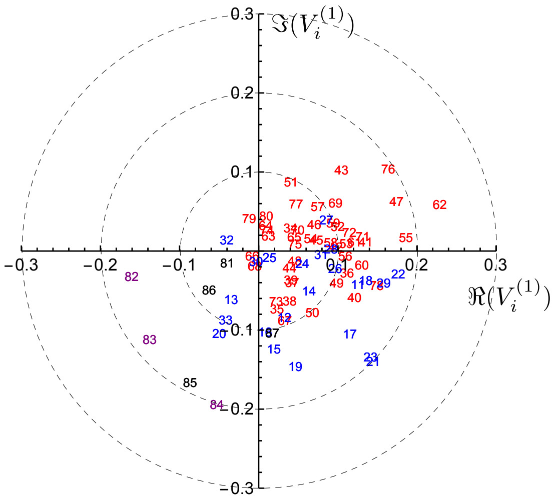

Figure S6: **Collective behavior of individual prices in the former half.** Eigenvector of the largest eigenvalue obtained for the former half of the whole period without the IPI prices, depicted in the complex plane.

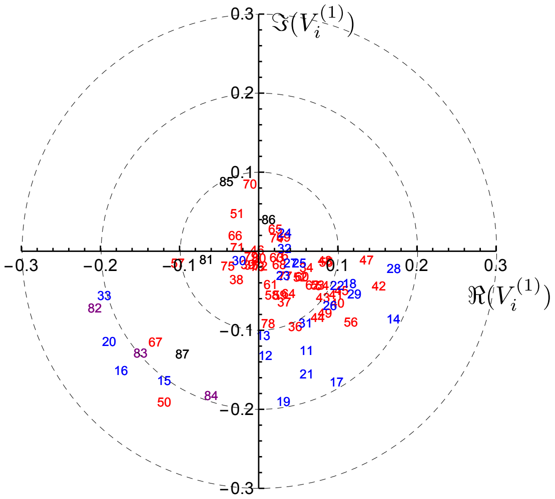

Figure S7: **Same as Fig. S6, but in the latter half.** Eigenvector of the largest eigenvalue obtained for the latter half of the whole period without the IPI prices, depicted in the complex plane.

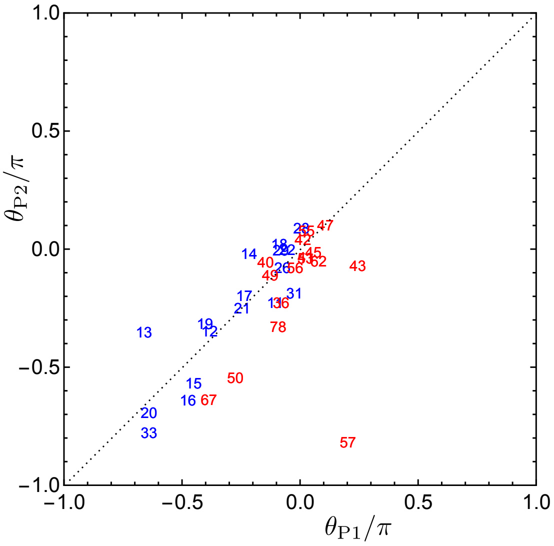

Figure S8: **Comparison of the lead-lag relations of the domestic prices between the two half periods.** The phase θ_{P2} of each of the domestic prices which are statistically significant in the first eigenmode in the latter half period is plotted against the corresponding phase θ_{P1} in the former half period.

Appendix D Hodge decomposition

In this appendix we describe the Hodge decomposition we use in the main body.

Let us denote the flow from node α to node β by $F_{\alpha\beta}$ in a directed network with N nodes. BY this definition, it is anti-symmetric, $F_{\alpha\beta} = -F_{\beta\alpha}$. The weight $w_{\alpha\beta}$ is defined by,

$$w_{\alpha\beta} = \begin{cases} 1 & \text{if } F_{\alpha\beta} \neq 0; \\ 0 & \text{otherwise} \end{cases} \tag{S11}$$

which is symmetric, $w_{\alpha\beta} = w_{\beta\alpha}$.

The Hodge decomposition aims to split the flow $F_{\alpha\beta}$ to two parts:

$$F_{\alpha\beta} = F^{(\mathrm{g})}_{\alpha\beta} + F^{(\mathrm{c})}_{\alpha\beta}. \tag{S12}$$

The first term $F^{(\mathrm{g})}_{\alpha\beta}$ is the *gradient flow*, which can be written is the following manner:

$$F^{(\mathrm{g})}_{\alpha\beta} = w_{\alpha\beta}\left(\phi_\alpha - \phi_\beta\right). \tag{S13}$$

In the above, ϕ_α is called the *Hodge potential* of the node α, that provides a measure of hierarchy of the nodes. The second term $F^{(\mathrm{c})}_{\alpha\beta}$ $(= -F^{(\mathrm{c})}_{\beta\alpha})$ is the *circular flow* that satisfies the following:

$$\sum_{\beta=1}^{N} F^{(\mathrm{c})}_{\alpha\beta} = 0 \quad (\alpha = 1, \cdots, N). \tag{S14}$$

The equations (S12)–(S14) may be solved as follows: First we rewrite Eq. (S14) by using Eq. (S12) and Eq. (S13):

$$\sum_{\beta=1}^{N} H_{\alpha\beta}\phi_\beta = F_{\alpha\beta} \quad (\alpha = 1, \cdots, N). \tag{S15}$$

$$H_{\alpha\beta} = \delta_{\alpha\beta}\sum_{\gamma=1}^{N} w_{\alpha\gamma} - w_{\alpha\beta} \tag{S16}$$

It is straightforward to prove that the matrix $H = (H_{\alpha\beta})$ has only one zeromode, which corresponds to the freedom of choosing the baseline of the Hodge potentials, as long as the network is weakly connected. This zeromode is a trivial one, due to the fact that only the differences $\phi_i - \phi_j$ appears in the definitions. Also, if the network is not weakly connected, there is no way of determining hierarchy of the nodes that belong to different connected components, as they should be. Since there are $N-1$ variables, Eq. (S15) has always a solution as soon as we fix the baseline of the Hodge potentials. Once we have $\{\phi_1.\phi_2.\cdots\phi_N\}$, the circular flow $F^{(\mathrm{c})}_{\alpha\beta}$'s are obtained from Eq. (S12).

Appendix E Percolation analysis of price clusters

The size distributions of clusters obtained with three different values of g_c, 0.33 (supercritical condition), 0.55 (critical condition), and 1.0 (subcritical condition), are shown in Fig. S9. We observe that the cluster size distribution at $g_c = 0.55$ has a power-law tail. For $g_c = 1.0$, extremely large clusters appear to make most of prices connect to each other. The cluster size distribution at $g_c = 0.33$ indicates that prices form a number of small clusters. These situations are more clearly visible in Figs.S10 and S11, corresponding to Fig.10 obtained nearly at the percolation transition.

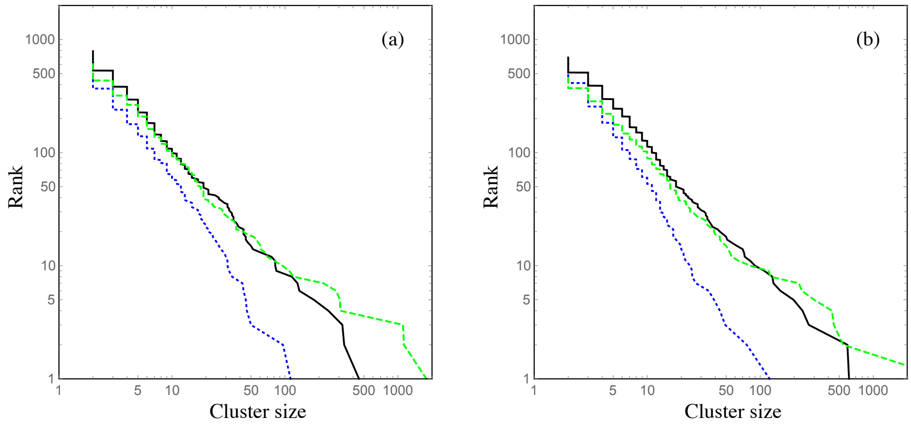

Figure S9: **Size distributions of price clusters for varied threshold g_c in the percolation model.** The panel (a) shows the results obtained at $g_c = 0.33$ (dashed green line), 0.55 (solid black line), and 1.0 (dotted blue line) for positive changes of domestic prices. The panel (b) shows the corresponding results for negative changes of domestic prices.

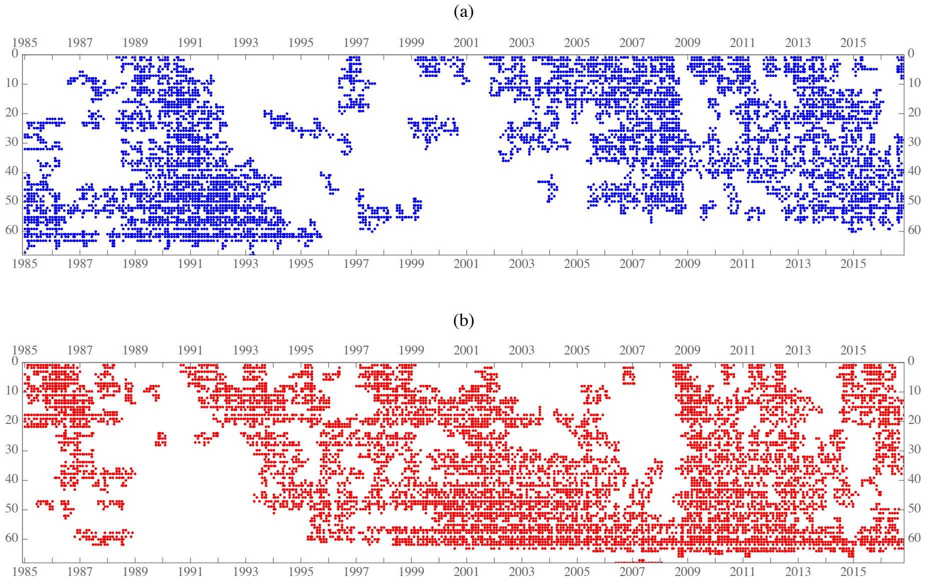

Figure S10: **Price clusters in a super-critical condition.** The panels (a) and (b) shows the 20 largest clusters as detected by the percolation analysis with $g_c = 0.3$, corresponding to the panels (a) and (b) in Fig. 10. .

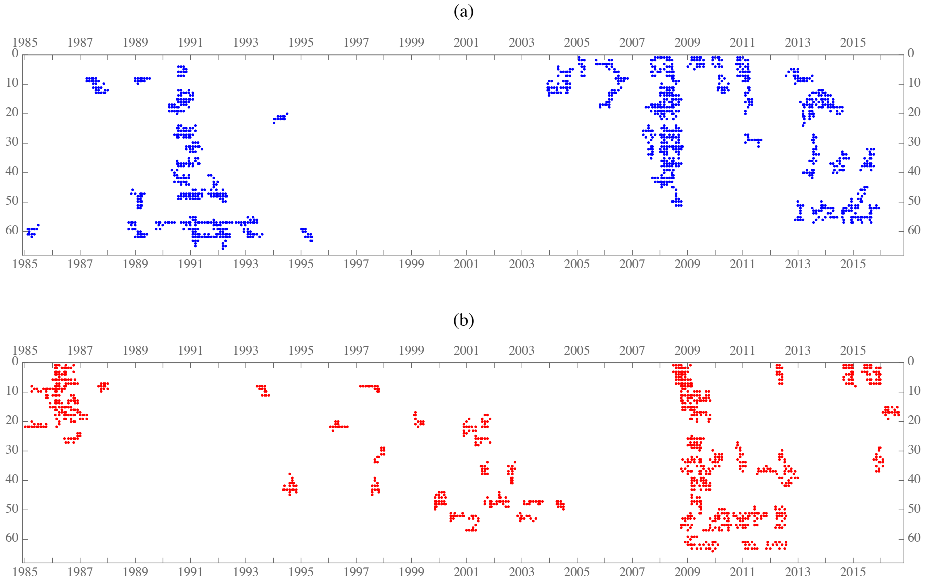

Figure S11: **Price clusters in a sub-critical condition.** Same as Fig. S10, but with $g_c = 1.0$.
